# Piezoelectric g-C_3_N_4_/rGO heterojunction remodels the periodontitis immune microenvironment to promote neurovascular–bone coupled osseointegration

**DOI:** 10.1016/j.mtbio.2026.103498

**Published:** 2026-07-27

**Authors:** Dan Li, Zhen Ai, Jinyang Lv, Yijun Zheng, Chao Zhang

**Affiliations:** Stomatological Hospital, School of Stomatology, Southern Medical University, Guangzhou, 510280, China

**Keywords:** Piezoelectric hydrogel, Heterojunction, Immunomodulation, Neurovascular–bone coupling, Osseointegration, Periodontitis

## Abstract

Dental implant restoration is required for tooth loss caused by periodontitis; however, the success rate of implantation in periodontitis patients is significantly lower than that in healthy individuals. On the one hand, biofilm colonization compromises early osseointegration of the implant; on the other hand, the chronic inflammatory microenvironment associated with periodontitis undermines long-term implant stability. Therefore, there is an urgent need for a strategy that integrates both antibacterial and osteogenic microenvironment-modulating functions to promote early osseointegration and long-term stability of dental implants. Herein, a two-dimensional nano-heterostructure was fabricated using g-C_3_N_4_ and rGO to construct a piezoelectric conductive hydrogel (CN/rGO) for implant restoration in periodontitis patients. Upon ultrasound stimulation, the CN/rGO hydrogel generates a piezoelectric field around the implant: in the early stage, it exerts antibacterial effects to promote initial osseointegration; over the long term, it modulates the immune microenvironment, thereby facilitating neurovascular regeneration and enhancing sustained osseointegration. Notably, inhibition of the JAK-STAT signaling pathway plays a pivotal role in CN/rGO-mediated macrophage phenotype regulation and reversal of the inflammatory microenvironment. This combined strategy of early piezoelectric antibacterial activity and long-term osteogenic microenvironment modulation holds significant implications for promoting early osseointegration and long-term stability of implants in periodontitis patients. The piezoelectric heterostructure-based design proposed in this study promotes neurovascular-guided bone regeneration around implants under inflammatory conditions, offering a feasible approach for improving implant success rates in periodontitis patients, preventing peri-implantitis, and enhancing the full-cycle service performance of dental implants.

## Highlights

• g-C_3_N_4_/rGO piezoelectric hydrogel enables short- and long-term synergistic therapy.
• Ultrasound-driven ROS generation provides immediate antibacterial protection.
• CN/rGO reprograms macrophage M1-to-M2 polarization via JAK-STAT inhibition.
• Immune remodeling drives integrated nerve–vessel–bone coupled regeneration.
• CN/rGO + US achieves robust osseointegration in a periodontitis implant model.

## Graphical abstract

**Figure:**
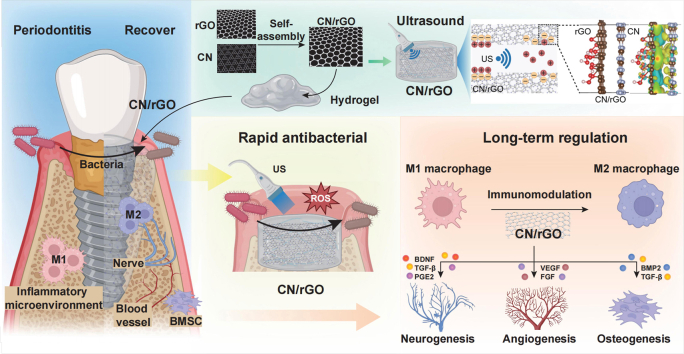

## 1 Introduction

Periodontitis, characterized by the destruction of periodontal supporting structures and subsequent tooth loss, adversely affects mastication and digestive absorption, and is ranked as the sixth most prevalent disease globally [1]. In China, severe periodontitis affects approximately 30.6% of adults aged ≥35, and 60%–70% of complete edentulism cases result from its progression, necessitating dental implant restoration [2]. However, the dysbiotic oral microbiota, localized biofilm accumulation, and chronic inflammatory microenvironment in periodontitis patients predispose them to inadequate early osseointegration, peri-implantitis, and even late-stage implant loosening [3]. Conventional treatments (antibiotics, scaling, bone augmentation) remain limited by incomplete biofilm removal and difficulty maintaining long-term alveolar bone height [4]. Unlike natural teeth, dental implants lack both a periodontal ligament and neural innervation, resulting in insufficient vascular and neural bone remodeling signals as well as the absence of occlusal force perception, which in turn compromises long-term osseointegration stability [5]. In natural teeth, mechanoreceptors in the periodontal ligament sense occlusal load and provide feedback that regulates masticatory force and alveolar bone remodeling [6]. Implants possess no periodontal ligament and little sensory innervation, so this feedback is largely lost; the reduced perception of load predisposes the site to occlusal overload and disturbs adaptive remodeling, a deficit often described as poor osseoperception [7]. Re-establishing sensory innervation around the implant is therefore relevant not only to sensation but to maintaining neuro-osteogenic coupling, supporting vascularization, and securing long-term stability. Furthermore, the inflammatory cascade within the periodontitis microenvironment not only impedes the osteogenic differentiation of bone marrow mesenchymal stem cells but also exacerbates neurovascular regeneration deficits in periodontal tissues [8]. Therefore, there is an urgent need for a strategy that simultaneously provides antibacterial activity, modulates the peri-implant immune microenvironment, and promotes neurovascular regeneration, thereby facilitating early osseointegration and long-term stability of implants in periodontitis patients.

Piezoelectric sonodynamic therapy (Piezoelectric-SDT) is a therapeutic approach that harnesses the piezoelectric potential and redox reactions generated by piezoelectric materials under external stimulation to efficiently produce electric fields and reactive oxygen species (ROS). This therapy exerts antibacterial effects through ROS generation [9], while the concomitant electric field modulates cellular voltage-gated ion channels and activates downstream signaling pathways, thereby regulating biological processes such as cell migration, proliferation, and differentiation, and ultimately promoting tissue repair [10]. Graphitic carbon nitride (g-C_3_N_4_, abbreviated as CN) and reduced graphene oxide (rGO) are both two-dimensional nanomaterials. CN possesses a relatively large band gap (≈2.6 eV) and favorable band edge potentials; however, it suffers from rapid charge recombination, and a paucity of active sites [11]. In contrast, rGO exhibits good hydrophilicity, excellent electrical conductivity, and a high affinity for neural cells [12]. By constructing a CN/rGO two-dimensional nano-heterostructure from these two components, electron–hole pair separation in CN can be effectively promoted under an external ultrasound field, significantly enhancing the catalytic performance and biological efficacy of the material. This in turn strengthens the antibacterial effect through ultrasound-responsive catalytic ROS generation and augments the regulation of the osseointegration microenvironment via piezoelectric properties, providing robust support for implant restoration in periodontitis patients.

**Scheme 1:**
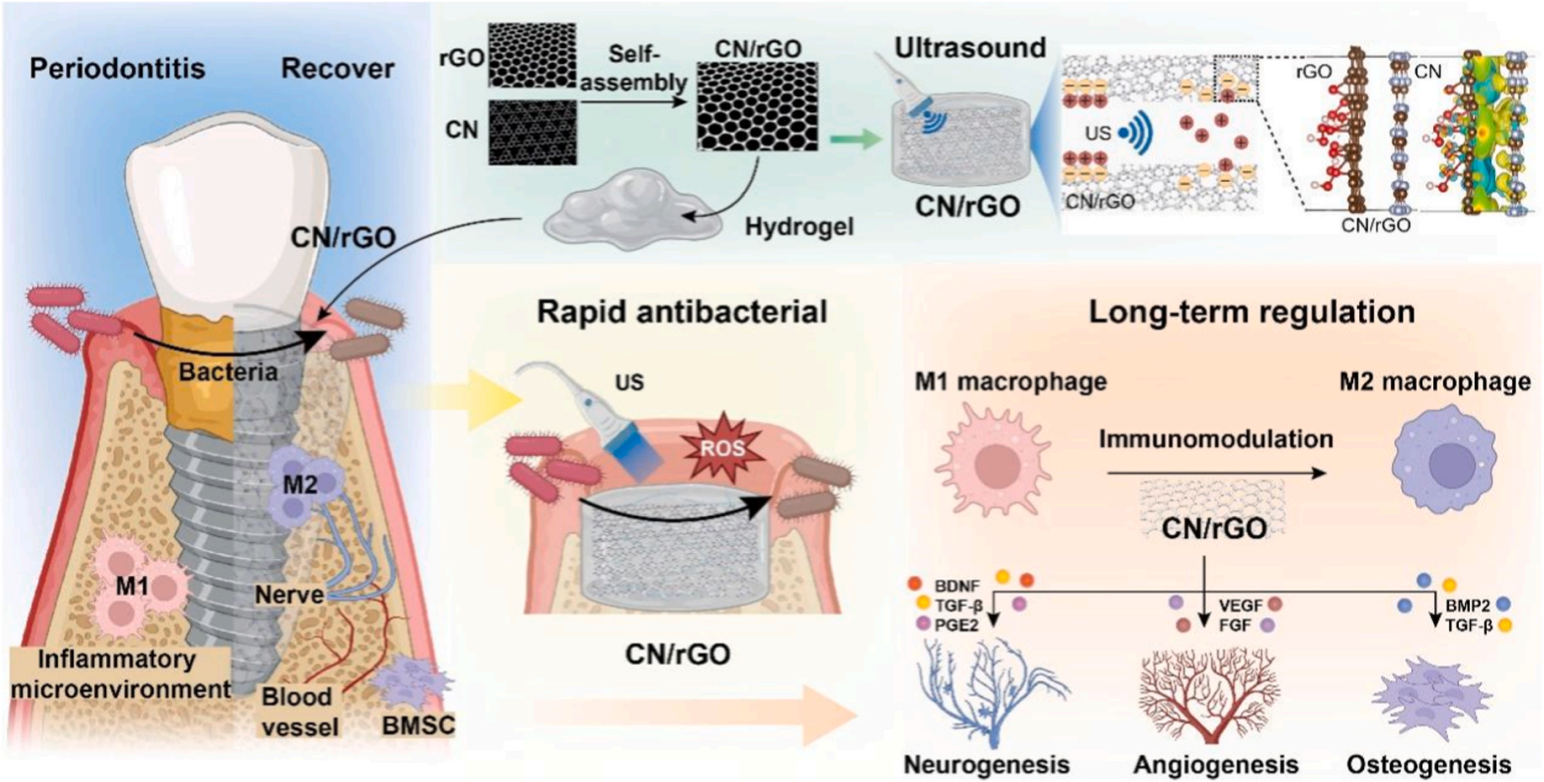
Schematic illustration of the piezoelectric activity of the CN/rGO conductive piezoelectric hydrogel and its regulation of the peri-implant osteogenic microenvironment in periodontitis through a “combined long- and short-term effect” strategy. 1) Immediate and highly efficient antibacterial action via the piezoelectric effect (short-term effect); 2) Induction of macrophage secretion of signaling molecules to modulate the periodontal osteogenic microenvironment, thereby enhancing vascular, neural, and bone interactions to promote osseointegration of dental implants (long-term effect).

On this basis, our study innovatively employs a conductive injectable hydrogel loaded with CN/rGO two-dimensional nanosheet heterostructures for dental implant restoration in periodontitis patients, aiming to simultaneously achieve antibacterial activity and osteogenic microenvironment modulation through piezoelectric sonodynamic therapy (Scheme 1). The core design advantage lies in the following: upon ultrasound stimulation, the heterostructure rapidly generates a piezoelectric field and ROS around the implant, exerting an immediate and highly efficient antibacterial effect (short-term effect); concurrently, it continuously induces macrophage secretion of relevant signaling molecules, enhancing neural perception, angiogenesis, and bone tissue interactions, thereby promoting osseointegration of dental implants (long-term effect). Notably, the inhibition of the JAK-STAT signaling pathway in macrophages by CN/rGO plays a pivotal role in modulating macrophage polarization phenotype and reversing the local chronic inflammatory state. This synergistic strategy of “rapid antibacterial action (short-term effect)–immunomodulation/tissue regeneration (long-term effect)" based on the piezoelectric sonodynamic effect of two-dimensional nano-heterostructures offers a novel therapeutic approach for modulating the peri-implant osseointegration microenvironment.

Many strategies have been developed to control peri-implantitis. Mechanical debridement and local or systemic antibiotics remain the clinical mainstay, but biofilm is difficult to remove completely and the use of antibiotics is increasingly constrained by resistance [13]. Antibacterial coatings that release silver, zinc, or copper ions, or antimicrobial peptides, provide durable bactericidal activity, but their dose cannot be adjusted once implanted and sustained ion release raises concerns about cytotoxicity [14]. Moreover, studies indicate that remodeling the inflammatory microenvironment is decisive for durable osseointegration [15,16]. The present system follows this direction: within a single injectable interface it combines early piezoelectric antibacterial action with later immunomodulation, and it brings macrophage-mediated neurovascular–bone coupling into the control of peri-implant osseointegration under periodontitis.

## 2 Results and discussion

### 2.1 Characterization of the piezoelectric heterostructure CN/rGO

To optimize the compositional ratio of the heterostructure, CN/rGO heterostructures with varying rGO contents (10 wt%, 15 wt%, and 20 wt%) were prepared, and their electrical conductivity and ROS generation capacity were evaluated (Fig. S1). Electrochemical impedance spectroscopy (EIS) results revealed that the charge transfer resistance (*R*_*ct*_) of the heterostructure progressively decreased with increasing rGO content, indicating a continuous improvement in electrical conductivity. Methylene blue (MB) degradation assays demonstrated that the 15 wt% rGO group exhibited the highest ·OH generation efficiency, whereas the 20 wt% group showed a decline, suggesting that excess rGO shielded the piezoelectric catalytic active sites of CN. Considering both parameters, the 15 wt% rGO content achieved an optimal balance between ROS catalytic efficiency and electrical conductivity; therefore, this ratio was adopted for all subsequent experiments.

**Fig. 1:**
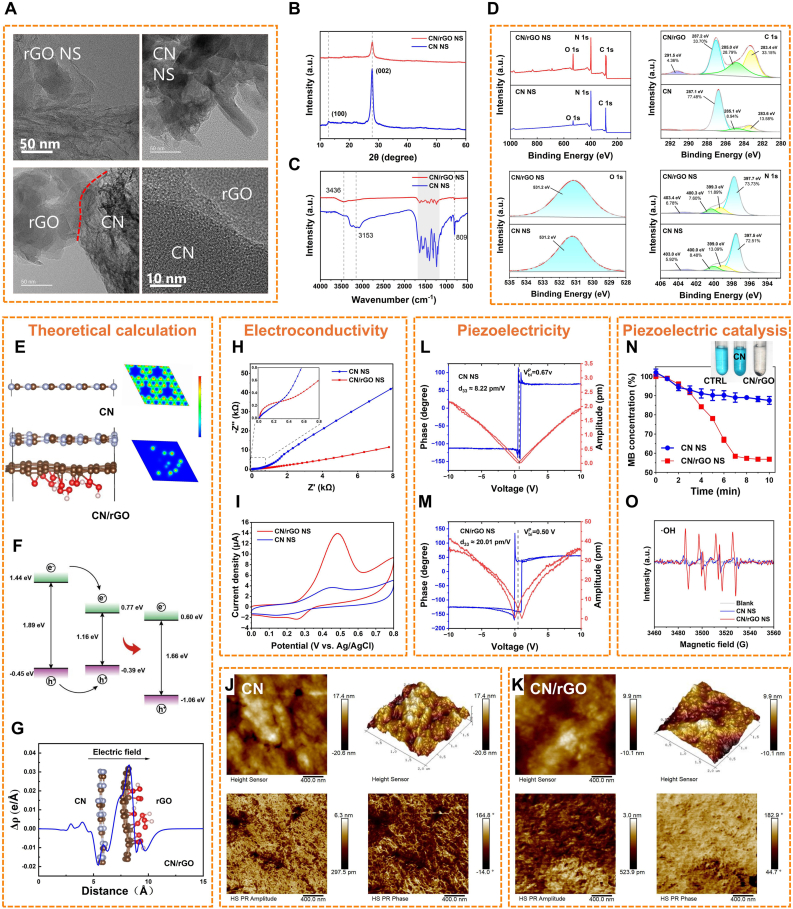
Characterization of CN/rGO. (A) Representative transmission electron microscopy (TEM) images of GO, CN, and CN/rGO heterostructure, and magnified images of the CN/rGO interfacial region. (B) X-ray diffraction (XRD) patterns of CN, rGO, and CN/rGO. (C) Fourier transform infrared (FT-IR) spectra of CN, rGO, and CN/rGO. (D) X-ray photoelectron spectroscopy (XPS) survey spectra and high-resolution C 1s, O 1s, and N 1s spectra of CN, rGO, and CN/rGO. First-principles calculations: (E) Optimized structural models and two-dimensional average charge density of CN and CN/rGO heterostructure. (F) Fermi energy level alignment of CN and rGO. (G) Planar-averaged charge density difference at the CN/rGO interface. (H) Electrochemical impedance spectroscopy (EIS) Nyquist plots of CN and CN/rGO (inset: equivalent circuit model). (I) C-V curves were recorded at a scan rate of 30 mV/s to monitor the electrode changes of CN/rGO and CN. Piezoresponse force microscopy (PFM) characterization: (J–K) Topography, piezoelectric amplitude, and phase images of CN and CN/rGO. (L–M) Amplitude butterfly loops and phase hysteresis curves of CN and CN/rGO. Piezoelectric catalytic performance: (N) Time-dependent methylene blue (MB) degradation rate of CN/rGO (0.1 mg/mL) under ultrasound irradiation. (O) Electron spin resonance (ESR) spectra of ·OH catalytically generated by CN and CN/rGO after ultrasound treatment (1.0 MHz, 2 W cm^−2^, 50% duty cycle, 5 min). (For interpretation of the references to color in this figure legend, the reader is referred to the Web version of this article.)

Based on the above optimal ratio (15 wt% rGO), the CN/rGO heterostructure was systematically characterized. Transmission electron microscopy (TEM) images revealed that the GO nanosheets exhibited a transparent, wrinkled fabric-like morphology, while the CN nanosheets displayed a dark, thick stacked-layer structure (Fig. 1A). In the CN/rGO heterostructure, a distinct interface between rGO and CN was observed. Magnified images revealed the formation of well-established interfacial contact [17]. X-ray diffraction (XRD) was employed to characterize the crystal structure of the heterostructure: characteristic peaks corresponding to the (100) and (002) diffraction planes of CN were identified at approximately 12.8° and 27.8°, with no peak shift observed, indicating that CN and rGO are primarily bonded through hydrogen bonds and van der Waals forces without disrupting the intrinsic structure of CN (Fig. 1B). This confirms that rGO is uniformly dispersed within the CN matrix, forming intimate interfacial contact conducive to electron transfer. In the Fourier transform infrared (FT-IR) spectrum, the triazine ring peak at 809 cm^−1^ and the fingerprint region at 1200–1650 cm^−1^ represent characteristic peaks of CN, none of which exhibited shifts (Fig. 1C). However, the peak at 3154 cm^−1^ (N–H) was attenuated while the peak at 3436 cm^−1^ (–OH) was intensified. This indicates O–H⋯N hydrogen bonding between the oxygen-containing groups of rGO and the amino/imino groups of CN, establishing a π-conjugated interface that promotes charge transport. In the XPS spectra, the π–π* satellite peak at 291 eV and the sp^2^ carbon characteristic peak at 285.0 eV in the C 1s spectrum suggest the formation and enhancement of the π-conjugated interface (Fig. 1D). The N 1s spectrum showed an increased C–N=C (397.7 eV) to N–(C)_3_ ratio, corroborating the FT-IR results. Based on the above analyses, the driving forces for the spontaneous assembly of CN and rGO are π–π stacking and weak hydrogen bonding; the flexible rGO network can protect CN and mitigate structural collapse of the piezoelectric phase under ultrasonication [18].

To gain deeper insight into the charge transfer (Fig. 1E–G), density-of-states (DOS) calculations show that rGO acts as a conductive medium forming a Schottky contact with g-C_3_N_4_, thereby promoting the separation of charge carriers [19]. Under ultrasound, the piezoelectric polarization field of g-C_3_N_4_ drives charge separation, while the Schottky contact and the conductive rGO network extract and transport electrons and suppress electron–hole recombination. This is consistent with the lower charge-transfer resistance (*R*_ct_, Fig. 1H) and the stronger ultrasound-driven current response. Cyclic voltammetry (CV) curves showed that the CN/rGO heterostructure exhibited a larger CV curve area compared with the CN electrode (Fig. 1I). These results collectively confirm that the electron transfer channel of the CN/rGO heterostructure accelerates charge separation, enhancing piezoelectric responsiveness. The piezoresponse of CN and CN/rGO was characterized by piezoresponse force microscopy (PFM). To limit electrostatic interference, the sample surface was leveled before measurement and the substrate was thoroughly grounded to reduce surface-charge accumulation and contact potential differences [20]. PFM revealed a high degree of consistency between the bright and dark regions in the amplitude and phase images under applied bias voltage (Fig. 1J and K). The butterfly-shaped amplitude curves demonstrated that the CN/rGO heterostructure underwent localized polarization switching under bias, with a significantly higher amplitude than that of CN, indicative of a stronger piezoelectric response (Fig. 1L and M). The addition of rGO enhanced the piezoresponse systematically while leaving the loop shape unchanged, without the irregular fluctuations expected from electrostatic contributions. The response therefore originates mainly from the intrinsic electromechanical coupling of the composite rather than from the conductive rGO network. Because contact-mode PFM of rGO-containing systems is susceptible to electrostatic artifacts, the amplitude data are used here only for semi-quantitative comparison between groups; the principal evidence for the piezoelectric activity of CN/rGO derives from the macroscopic measurements described below, which are not subject to these artifacts.

To verify the piezoelectric catalytic performance of the heterostructure under ultrasonication, capture experiments were conducted using ROS-specific indicators. Hydroxyl radicals (·OH), a highly corrosive ROS species, can bleach methylene blue (MB) to a colorless state. Detection using CN/rGO heterostructure dispersions at various concentrations revealed that ·OH generation capacity reached a plateau above a concentration of 0.1 mg/mL (Fig. S2). The time-dependent ·OH generation of the 0.1 mg/mL heterostructure dispersion was further examined: after 5 min of ultrasonication, the absorbance of the MB solution decreased by half, indicating extremely potent redox catalytic capability (Fig. 1N). Electron spin resonance (ESR) measurements also demonstrated that after 5 min of ultrasonication, the CN group produced only a weak ·OH signal, whereas the heterostructure group displayed a strong ·OH signal, confirming its capacity to catalytically generate substantial amounts of ·OH in response to ultrasound stimulation (Fig. 1O). These reactive oxygen species are the catalytic product of ultrasound-driven piezoelectric charge separation and dominate the early antibacterial phase, whereas the persistent piezoelectric field provides the electrical signal for macrophage immunomodulation. The ESR and methylene blue assays above confirm that the material can generate reactive oxygen species under ultrasound stimulation. CCompared with other piezoelectric systems, CN/rGO offers several practical advantages. It is metal-free, which avoids the Ba^2+^/Zn^2+^ release and associated toxicity of BaTiO_3_ or ZnO [21] and distinguishes it from MoS_2_- or BiOCl-based materials [22]. Its π-conjugated conductive rGO interface promotes charge separation and is also favorable for neural cells, while the injectable conductive hydrogel form conforms to the irregular peri-implant defect. Whereas most related work addresses antibacterial action or a single piezoelectric stimulus, the present design targets coupled immune–neurovascular–bone remodeling of the osteogenic microenvironment (Table S2).

### 2.2 Preparation and characterization of CN/rGO hydrogel

**Fig. 2:**
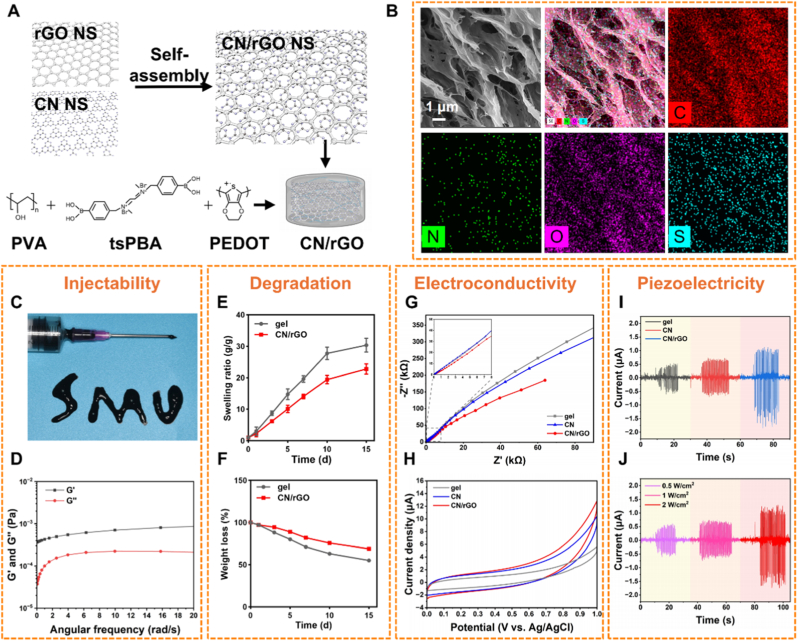
Physicochemical properties of the CN/rGO piezoelectric conductive hydrogel. (A) Schematic illustration of the hydrogel preparation process. (B) Scanning electron microscopy (SEM) images showing the porous microstructure of the hydrogel scaffold. (C) Injectability demonstration through a 0.9 × 38 mm needle. (D) Frequency sweep rheological testing at 37°C, showing the storage modulus (G′) and loss modulus (G″). (E) Swelling ratio of gel and CN/rGO hydrogel over time. (F) Enzymatic degradation curves of gel and CN/rGO hydrogel over 15 days. (G) EIS Nyquist plots and (H) cyclic voltammetry (CV) curves of gel and CN/rGO hydrogel. (I) Piezoelectric current output of gel, CN-containing hydrogel, and CN/rGO hydrogel at different ultrasound intensities. (J) Piezoelectric current output of CN/rGO hydrogel at different ultrasound intensities.

A conductive hydrogel (gel) containing poly(3,4-ethylenedioxythiophene) (PEDOT) was used to self-assemble and load CN/rGO, yielding a conductive piezoelectric hydrogel (hereafter referred to as CN/rGO) (Fig. 2A). Because the hydrogel must fill the gap between the implant and the socket bone wall, injectability and in situ self-healing are the primary considerations in its design. The matrix is a dynamic network formed by polyvinyl alcohol (PVA, 6 wt%) and the phenylboronic acid cross-linker tsPBA (3 wt%) through boronate ester bonds: the former provides a biocompatible backbone and vicinal diol groups, while the latter serves as the cross-linker. These reversible bonds endow the gel with injectability, self-healing, and responsiveness to pH and reactive oxygen species (ROS) [23]. PEDOT serves as the conductive phase, providing electron-transport channels and coupling with the piezoelectric output of the embedded CN/rGO. Compared with conventional hydrogels, this system permits minimally invasive needle injection and achieves a tight, in situ fit at the irregular extraction-socket interface; after mechanical deformation, it autonomously reconstructs its network to accommodate the changing geometry and loading conditions around the implant. To determine the optimal loading concentration of CN/rGO heterostructure within the hydrogel, hydrogels with varying CN/rGO contents (0.25 wt%, 0.5 wt%, and 1.0 wt%) were prepared and comprehensively screened from two perspectives: ROS generation and cytocompatibility (Fig. S3). The results showed that ROS generation capacity increased with CN/rGO content but plateaued beyond 0.5 wt%. CCK-8 assay results indicated that macrophage viability was maintained above 90% at CN/rGO contents ≤0.5 wt%, whereas a significant decline in viability was observed in the 1.0 wt% group, possibly attributable to the aggregation of two-dimensional nanosheets at high concentrations. Therefore, considering both functional efficacy and biosafety, 0.5 wt% was selected as the optimal loading concentration for subsequent experiments. All subsequent characterization results are based on the 0.5 wt% CN/rGO hydrogel.

The microstructure of the hydrogel scaffold revealed that CN/rGO possessed a uniform pore size distribution (Fig. 2B). The hydrogel could be smoothly extruded through a 0.9 × 38 mm needle without clogging, confirming its excellent injectability (Fig. 2C). Rheological testing showed that during frequency sweeps, the storage modulus (G′) of the hydrogel consistently exceeded the loss modulus (G″) under oscillatory stress; during amplitude sweeps under oscillatory shear, the moduli remained unchanged over time with G′ consistently exceeding G'' (Fig. 2D–S4). Meanwhile, its viscosity decreased markedly with increasing shear rate, indicating that the dynamic hydrogel network conferred shear-thinning behavior and injectability (Fig. S5). These findings suggest that this system is a structurally stable, elasticity-dominated, shear-thinning hydrogel. The swelling ratio and degradation performance of the hydrogel were evaluated at different time points. Compared with the gel group, the swelling ratio of CN/rGO decreased to 22.77% after 12 h, indicating that the incorporation of the CN/rGO heterostructure enhanced the structural stability of the hydrogel, thereby preventing mechanical deterioration and rapid loss of active components caused by excessive swelling, making it more suitable for the in vivo microenvironment of implant restoration in periodontitis (Fig. 2E). Weight loss tests showed 31.25% degradation within 15 days (Fig. 2F).

To evaluate the maintenance of piezoelectric catalytic function during degradation, the CN/rGO hydrogel was immersed in phosphate-buffered saline (PBS) containing lysozyme to simulate the in vivo degradation environment. The MB catalytic degradation rate under ultrasonication was measured on days 1, 3, 7, and 14 to indirectly reflect the temporal maintenance of piezoelectric catalytic activity (Fig. S6). Using the day 1 MB degradation rate as a baseline, the results showed that relative catalytic activity remained at a relatively high level on days 3 and 7, suggesting that the loosening of the hydrogel network structure during later stages of degradation weakened the effective immobilization of the CN/rGO heterostructure. Additionally, the piezoelectric current output under ultrasonication was directly measured for the hydrogel on days 1 and 14 (Fig. S6), revealing a significant decrease in piezoelectric current on day 14 compared with day 1. These results indicate that during the early phase of hydrogel degradation (approximately within 2 weeks), the CN/rGO heterostructure remains effectively immobilized within the hydrogel network and can continuously generate piezoelectric fields and ROS under ultrasound stimulation. Although the boronate ester bonds can be oxidatively cleaved, ultrasound is applied intermittently, so reactive oxygen species (ROS) are generated in pulses rather than released continuously, and the reversible bonds allow the network to re-heal during the intervals between treatments [24]. Consistent with this, the piezocatalytic activity and piezoelectric current were largely retained through day 7 and declined only by day 14 (Fig. S6), while the mass loss proceeded progressively (Fig. 2E and F). Degradation tests in PBS and in PBS containing lysozyme showed that the network remains functional over an antibacterial and immunomodulatory window of approximately two weeks before degrading and being replaced by new tissue.

Electrochemical impedance and CV curves (Fig. 2G and H) showed that, compared with the gel group, CN/rGO exhibited lower impedance, higher ionic conductivity, and CV curves with a larger enclosed area and higher oxidation peaks, indicating enhanced electron conduction and storage capacity. A light-emitting diode (LED) was illuminated by connecting a circuit through the hydrogel, visually confirming its excellent electrical conductivity (Fig. S7). With increasing ultrasound intensity, the piezoelectric output performance of the CN/rGO-loaded hydrogel was significantly enhanced, providing the electric field foundation for the “short-term effect” antibacterial action (Fig. 2I and J). The intrinsic piezoelectric/piezocatalytic response of CN/rGO is supported by macroscopic evidence independent of electrostatic effects—specifically, the directly measured ultrasound-driven piezoelectric short-circuit current and the ultrasound-dependent catalytic ROS generation. Furthermore, hemolysis assay results showed no significant difference in hemolysis rates between the gel and CN/rGO groups compared with the PBS group (Fig. S8). Subcutaneous implantation experiments demonstrated that the CN/rGO hydrogel gradually degraded within 2 weeks (Fig. S9), and hematoxylin and eosin (H&E) staining confirmed that it caused no apparent histological damage to major organs in rats (Fig. S10), fully demonstrating the excellent biocompatibility and biodegradability of the CN/rGO hydrogel.

### 2.3 Bactericidal activity and antibacterial mechanism of the conductive piezoelectric sonosensitive hydrogel

**Fig. 3:**
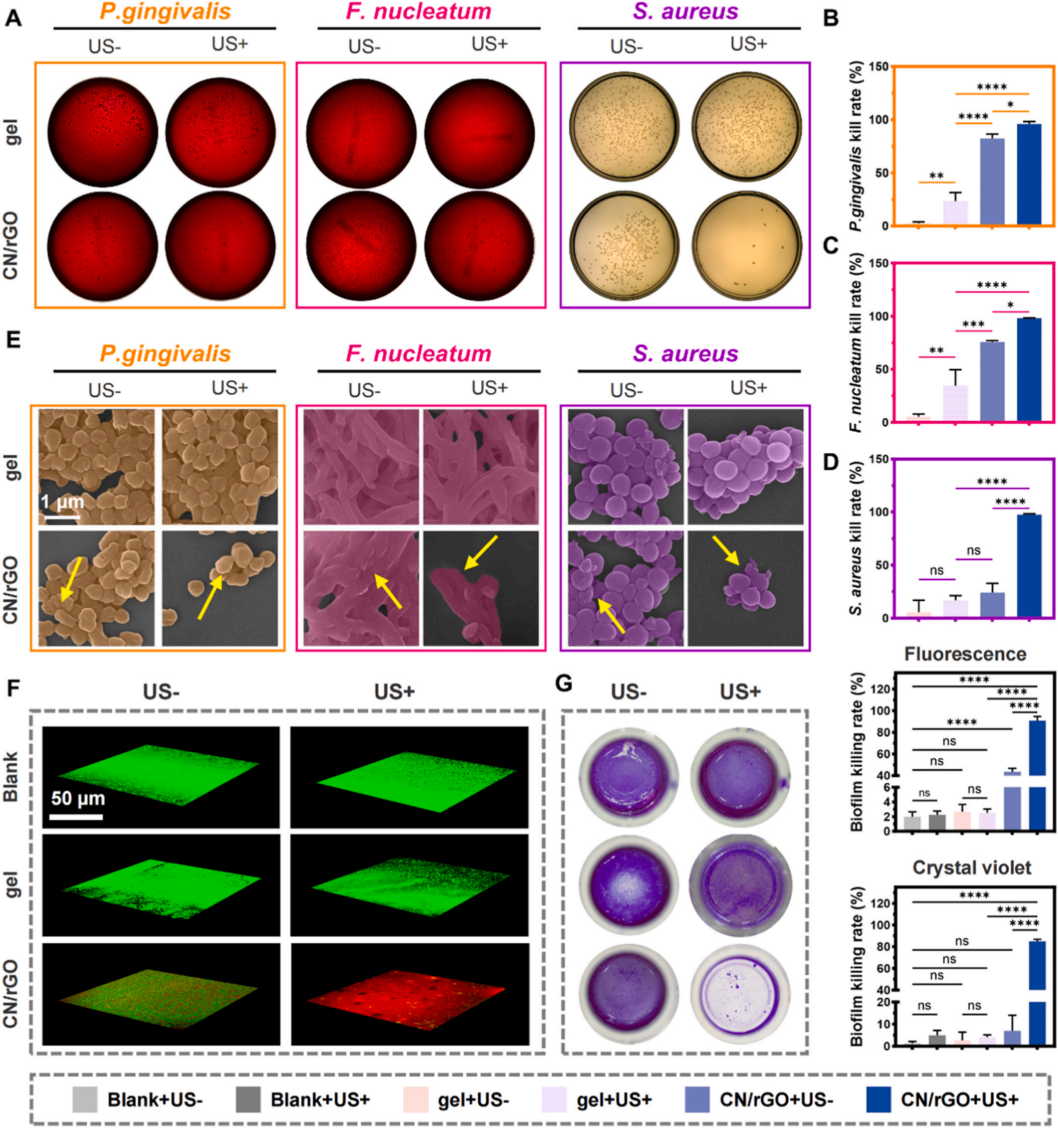
Antibacterial effects of CN/rGO hydrogel against periodontitis-associated pathogens. (A) Representative colony-forming unit (CFU) plate images of *Porphyromonas gingivalis* (*P. gingivalis*), *Fusobacterium nucleatum* (*F. nucleatum*), and *Staphylococcus aureus* (*S. aureus*) after treatment in each group. (B) Quantitative CFU analysis of *P. gingivalis*, (C) *F. nucleatum*, and (D) *S. aureus*. (E) SEM images of bacterial morphology after treatment in each group. (F) Live/dead fluorescence staining of multi-species biofilms under conditions without ultrasound (US−) and with ultrasound (US+). (G) Crystal violet staining for quantification of total biofilm biomass. US− = without ultrasound; US+ = with ultrasound (1.0 MHz, 1.5 W cm^−2^, 50% duty cycle, 5 min). Data are means ± SD (n = 3). One-way ANOVA or Two-way ANOVA was followed by Dunnett's test. *P < 0.05, **P < 0.01, ***P < 0.001, ****P < 0.000, ns = not significant. (For interpretation of the references to color in this figure legend, the reader is referred to the Web version of this article.)

Bacterial biofilm colonization is the initiating factor and central pathogenic element in both periodontitis and peri-implantitis. *Porphyromonas gingivalis*, *Fusobacterium nucleatum*, and *Staphylococcus aureus* are specific pathogens associated with peri-implantitis. Colony-forming unit (CFU) assay results showed that the gel group exhibited no appreciable antibacterial effect regardless of whether ultrasound was applied. In contrast, CN/rGO exerted potent antibacterial effects under ultrasound stimulation (Fig. 3A–D); even without ultrasound, a mild antibacterial effect was observed (p < 0.001), which may be attributed to physical cutting by the edges of the CN/rGO heterostructure nanosheets, leading to bacterial cytoplasmic leakage. SEM images (Fig. 3E) showed that all three bacterial species in the control group maintained normal morphology, with some cells in a dividing state indicative of the proliferative phase, whereas CN/rGO under ultrasonication induced bacterial membrane rupture and cytoplasmic leakage. These findings confirm that the antibacterial effect is associated with ultrasound-triggered piezoelectric catalytic ROS generation.

Dental plaque biofilm is the initiating factor in the onset and progression of periodontitis and peri-implantitis. Extracellular polysaccharides secreted by bacteria form a protective matrix that impedes the penetration of antimicrobial agents. Mixed-species biofilms formed by co-culturing the three bacterial species more closely approximate the clinical microenvironment in vitro. Live/dead staining results (Fig. 3F) showed that the gel-treated group exhibited abundant green, thick biofilm plaque on the surface, whereas after CN/rGO treatment with ultrasound, biofilm thickness was reduced and the surface was almost entirely covered by red (dead) bacteria, with a bacterial killing rate of approximately 90.79%. Crystal violet quantification at OD 600 nm (Fig. 3G) demonstrated that biofilm mass in the US + CN/rGO group was significantly reduced, with an anti-biofilm rate exceeding 80%. These results suggest that the piezoelectric field enhanced the penetration capacity of ROS, disrupting the extracellular polysaccharide barrier of the biofilm and achieving deep-seated sterilization. CN/rGO, through ultrasound-triggered “piezoelectric–ROS” cascade reactions, can rapidly penetrate biofilms and disrupt bacterial membrane structures, eliminating bacteria and biofilms.

The above results confirm that the CN/rGO hydrogel, upon ultrasound activation, can efficiently eradicate pathogenic bacteria and biofilms, achieving early infection control around the implant (“short-term effect”). However, the long-term success of implant restoration in periodontitis patients depends not only on early antibacterial activity but, more critically, on the reversal of the chronic inflammatory microenvironment and subsequent tissue regeneration. Following implant placement, macrophages are the first immune cells to contact the material, and their polarization phenotype directly determines the trajectory of the local immune microenvironment, regulating downstream neurogenesis, angiogenesis, and osteogenic differentiation through paracrine effects [[25], [26], [27]]. Therefore, we next systematically evaluated the regulatory effects of CN/rGO hydrogel on macrophage polarization and progressively elucidated the “long-term effect” mechanism by which it mediates neurovascular–osteogenic coupling through immunomodulation.

### 2.4 CN/rGO hydrogel modulates the immune microenvironment

**Fig. 4:**
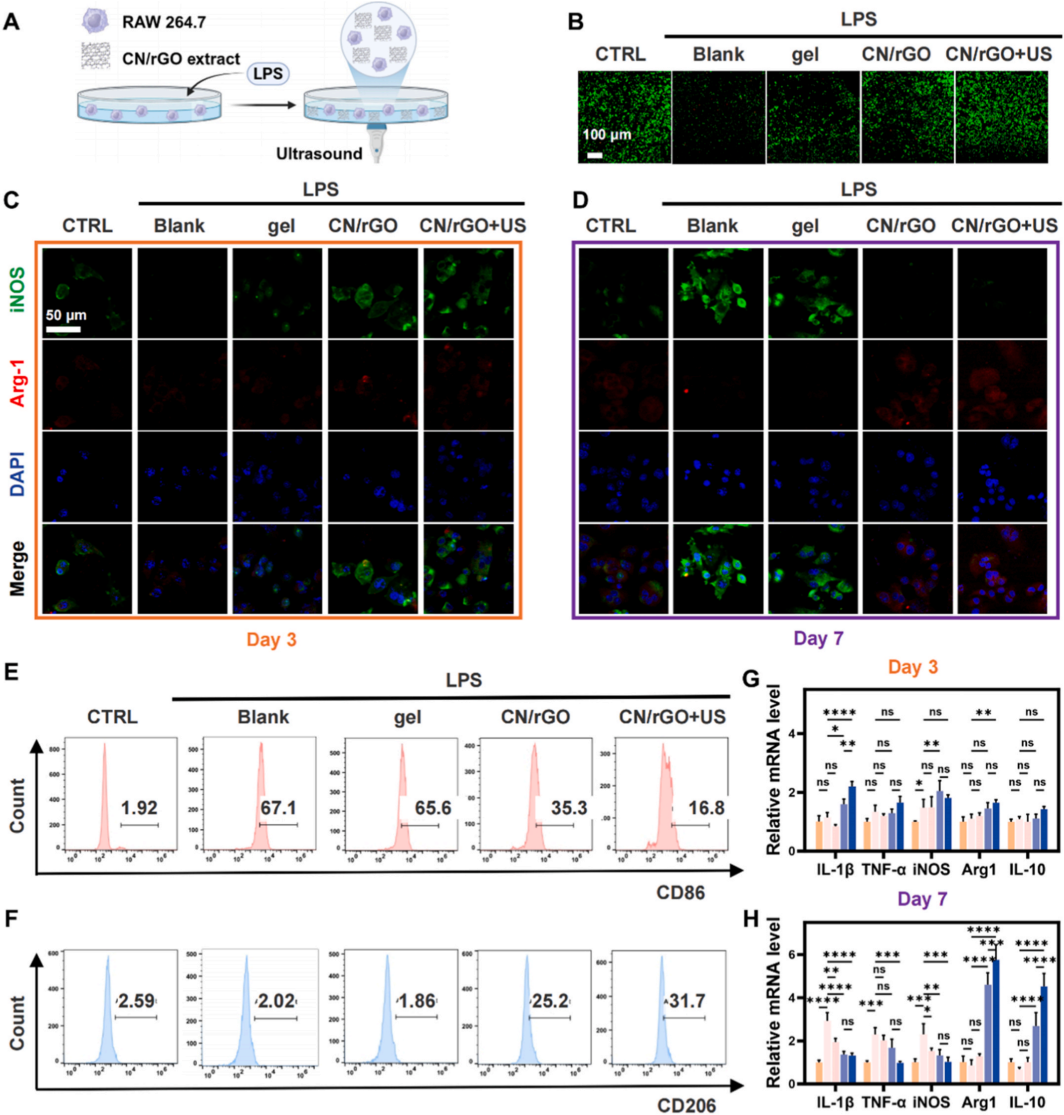
CN/rGO hydrogel modulates macrophage polarization under LPS-induced inflammatory conditions. (A) Schematic illustration of the experimental design. (B) Live/dead staining of RAW 264.7 macrophages cultured on different hydrogel surfaces for 3 days. Immunofluorescence co-staining of iNOS (M1 marker, green) and Arg-1 (M2 marker, red): (C) day 3 and (D) day 7, with corresponding fluorescence intensity quantification. Flow cytometry analysis of (E) CD86^+^ (M1) and (F) CD206^+^ (M2) macrophage proportions, and (G) quantification of the M1/M2 ratio. RT-qPCR detection of (H) pro-inflammatory gene (TNF-α, IL-6, IL-1β) and (I) anti-inflammatory gene (Arg-1, IL-10) expression levels on days 3 and 7. Data are means ± SD (n = 3). One-way ANOVA or Two-way ANOVA was followed by Dunnett's test. *P < 0.05, **P < 0.01, ***P < 0.001, ****P < 0.000, ns = not significant. (For interpretation of the references to color in this figure legend, the reader is referred to the Web version of this article.)

Bone regeneration does not depend on a single macrophage phenotype but rather on an orderly M1-to-M2 transition: the early M1 phase clears debris and bacteria while recruiting reparative cells, and the later M2 phase secretes pro-angiogenic and pro-osteogenic factors [28]. On this basis, the CN/rGO hydrogel exploits the ROS generated by early piezocatalysis to drive an antibacterial M1 phase and a sustained later-stage piezoelectric field to drive the M2 regenerative phase. To investigate the immunomodulatory effects of CN/rGO hydrogel on the inflammatory microenvironment, an in vitro inflammation model was established by stimulating RAW 264.7 macrophages with lipopolysaccharide (LPS) (Fig. 4A). In this assay, the macrophages were cultured directly on the hydrogel surface LPS was selected as the sole stimulatory factor because, in the periodontitis microenvironment, LPS released by Gram-negative periodontal pathogens (e.g., *P. gingivalis*) is the predominant pathological driver of macrophage M1 polarization, more closely recapitulating the actual pathogenic scenario of peri-implantitis. Live/dead cell staining (Fig. 4B) and CCK-8 assay (Fig. S11) results showed that LPS stimulation significantly inhibited cell proliferation in the gel group. In contrast, cells cultured on the CN/rGO hydrogel surface for 3 days exhibited favorable growth regardless of whether ultrasound was applied. Moreover, the ultrasound-treated group showed slightly enhanced cell proliferation, suggesting that brief ultrasound stimulation is non-cytotoxic and may modestly promote proliferation.

Following dental implant placement, macrophages immediately adhere to the surface and establish the critical early immune microenvironment^13^. Immunofluorescence results showed that iNOS expression in the LPS group was elevated compared with the blank control on day 3, indicating macrophage polarization toward the M1 phenotype (Fig. 4C–S12). The CN/rGO + US group exhibited further upregulation of iNOS expression relative to the LPS group; this is presumably because low-concentration ROS generated by ultrasound-activated CN/rGO transiently upregulated pro-inflammatory factor expression, enhancing M1 phenotype polarization. This transient M1 enhancement may assist in clearing colonizing bacteria during the early phase. By day 7, the LPS group still maintained elevated iNOS levels, indicating an excessive inflammatory response, whereas the CN/rGO + US group showed decreased iNOS expression and upregulated Arg-1 expression levels (Fig. 4D–S12), demonstrating a macrophage phenotypic switch toward the M2 phenotype and the initiation of tissue repair processes. Flow cytometry results revealed that the proportion of CD206^+^ cells in the CN/rGO + US group was significantly higher than that in the gel group (Fig. 4E–G), confirming that CN/rGO under ultrasound stimulation can continuously induce the transition of RAW macrophages toward the M2 phenotype. Ultrasound alone, without an accompanying piezoelectric material, had almost no effect on bacteria or on immunomodulation and was comparable to the untreated control, indicating that the effects observed here arise mainly from the piezoelectric output of CN/rGO rather than from ultrasound itself [29]. PCR results simultaneously verified this temporal polarization switch: on day 7, the expression of pro-inflammatory factors (TNF-α, IL-6, IL-1β) was downregulated, while the expression of anti-inflammatory factors (Arg-1, IL-10) was upregulated (Fig. 4H and I). Taken together, CN/rGO + US can relieve local immunosuppression in periodontitis by promoting macrophage M1 polarization through ROS in the early phase to clear pathogens, and subsequently inducing M2 polarization to reverse the inflammatory microenvironment, thereby laying the foundation for osseointegration.

### 2.5 Mechanism by which CN/rGO hydrogel modulates the immune microenvironment

**Fig. 5:**
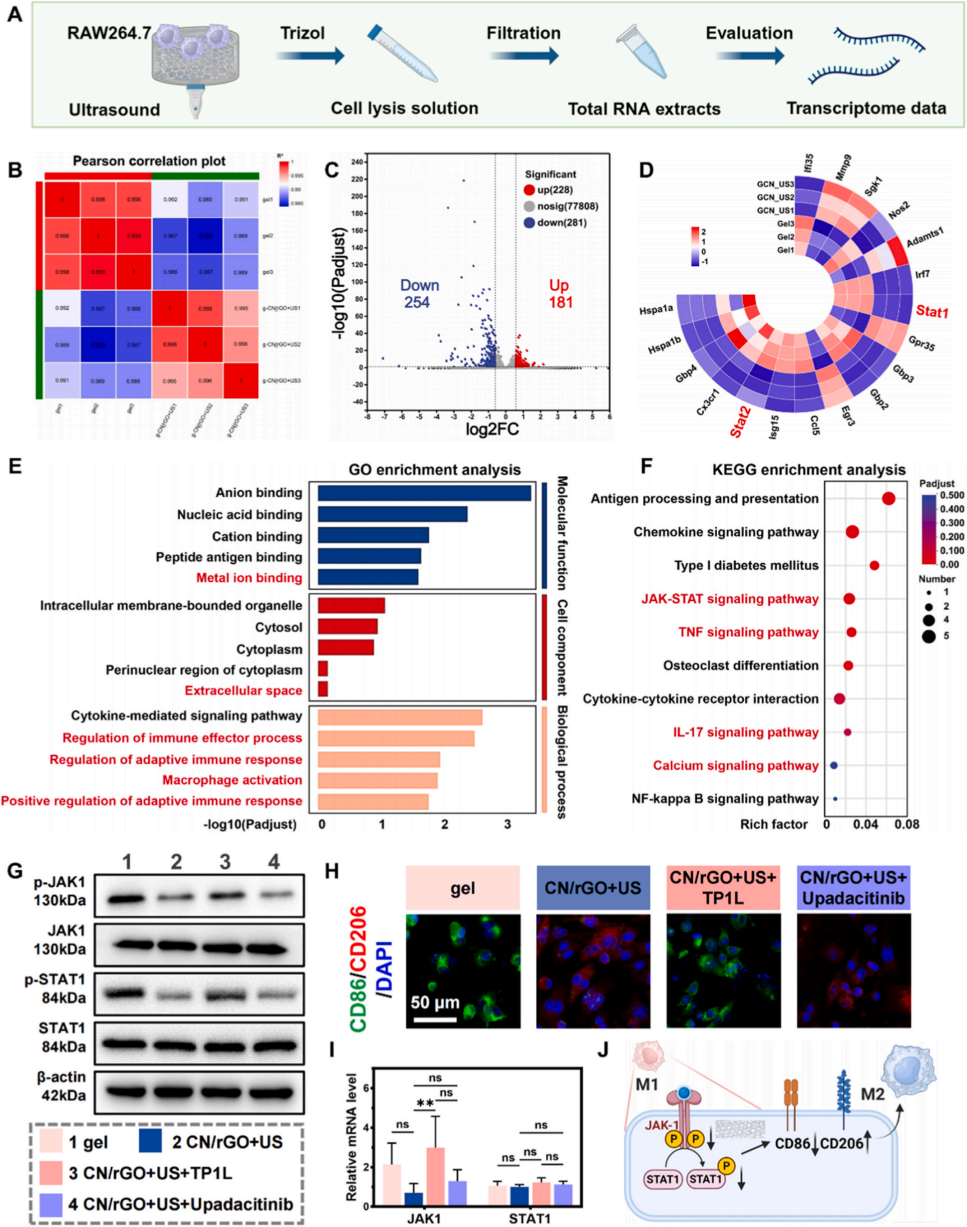
Transcriptomic analysis and functional validation of the JAK-STAT signaling pathway in CN/rGO + US-mediated macrophage immunomodulation. (A) Schematic illustration of the RNA-seq experimental workflow. (B) Heatmap of representative DEGs. (C) Volcano plot of differentially expressed genes (DEGs) between the CN/rGO + US group and the control group. (D) Downregulated expression profile of key pro-inflammatory and tissue regeneration-inhibitory genes in the CN/rGO + US group. (E) Gene Ontology (GO) enrichment analysis of DEGs. (F) Kyoto Encyclopedia of Genes and Genomes (KEGG) pathway enrichment analysis, highlighting significant changes in the JAK-STAT signaling pathway. (G) Western blot analysis of p-JAK1, JAK1, p-STAT1, and STAT1 protein expression levels in macrophages treated with JAK1 activator TP1L or JAK1 inhibitor Upadacitinib. (H) Immunofluorescence staining of CD86 (M1) and CD206 (M2) in macrophages before and after TP1L/Upadacitinib treatment. (I) RT-qPCR validation of JAK1 and STAT1 mRNA expression levels. (J) Schematic illustration of the mechanism by which CN/rGO + US promotes macrophage M2 polarization through inhibition of the JAK-STAT pathway. Data are means ± SD (n = 3). One-way ANOVA or Two-way ANOVA was followed by Dunnett's test. *P < 0.05, **P < 0.01, ***P < 0.001, ****P < 0.000, ns = not significant.

The above results demonstrated that CN/rGO + US can effectively regulate the temporal sequence of macrophage polarization; however, the underlying molecular mechanism remained unclear. To elucidate the core signaling pathway through which CN/rGO mediates immunomodulation to promote neurovascular–osteogenic coupling, transcriptomic analysis was performed on RAW cells treated with CN/rGO + US under LPS stimulation (Fig. 5A). Volcano plots revealed 181 upregulated genes and 254 downregulated genes in the CN/rGO + US group compared with the control (Fig. 5B and C). Among these, genes associated with pro-inflammatory responses (Gpr35, Stat1/2, Gbp2/3/4) and those involved in the inhibition of osteogenesis, angiogenesis, and neurogenesis (Egr3, Cx3cr1, Ccl5, Sgk1, Adamts1, Hspa1a/b, etc.) were all significantly downregulated (Fig. 5D). Gene Ontology (GO) enrichment analysis of differentially expressed genes (DEGs) revealed that biological processes, cellular components, and molecular functions were primarily enriched in inflammatory response, immune processes, and extracellular space-related pathways (Fig. 5E). Kyoto Encyclopedia of Genes and Genomes (KEGG) enrichment analysis indicated that the JAK-STAT, TNF, and IL-17 signaling pathways were all significantly altered in the CN/rGO + US group compared with the control, with the JAK-STAT signaling pathway showing the most pronounced changes (Fig. 5F).

The JAK-STAT signaling pathway is an evolutionarily highly conserved and structurally concise cytokine-to-nuclear transcription rapid transduction pathway. Gain-of-function and loss-of-function experiments were conducted using a specific JAK1 activator (TP1L) and inhibitor (Upadacitinib). PCR and Western blotting results showed that both the transcriptional levels and phosphorylation levels of JAK1 and STAT1 (p-JAK1 and p-STAT1) in macrophages of the CN/rGO + US group were significantly reduced (Fig. 5G–I). Immunofluorescence results revealed that macrophages in the CN/rGO + US + TP1L group exhibited decreased CD206 expression and increased CD86 expression, indicating that JAK1 activation can substantially counteract the M2 polarization effect induced by CN/rGO + US; upon addition of the inhibitor Upadacitinib, the M2 polarization effect was restored (Fig. 5H). In summary, the JAK-STAT signaling pathway represents the core mechanism through which CN/rGO + US promotes macrophage M2 polarization and exerts immunomodulatory effects. JAK1 phosphorylation is itself shaped by several upstream inputs: the piezoelectric field may act through changes in membrane potential and voltage-gated Ca^2+^ channels [30]; transient ROS can modify cysteine residues on JAK1 and its phosphatases, thereby altering its phosphorylation state [31]; and the mechanical component of ultrasound may engage mechanosensitive pathways [32]. From these observations and the literature, we propose an “ultrasound → piezoelectric field/ROS → (Ca^2+^ redox) → JAK1″ link as a working hypothesis, with the relative contribution of each input remaining to be defined. The M1-to-M2 transition reflects the combined action of multiple factors over time. In the early phase, high local concentrations of ROS, together with the piezoelectric field, drive a transient M1 state that clears bacteria. As the biofilm is cleared and pro-inflammatory stimulation wanes, the persistent piezoelectric field exerts a cumulative inhibitory effect on JAK-STAT signaling which, combined with the decay of ROS and the IL-10/Arg-1 feedback from the M2 cells themselves, reprograms macrophages toward the M2 phenotype by day 7. This in vitro timeline (days 3 to 7) falls within both the in vivo ultrasound window (days 1–14) and the period over which the material remains piezoelectrically active; once ultrasound is withdrawn on day 15, the established M2 microenvironment continues to support regeneration through paracrine signaling. Having established this mechanism, subsequent experiments were designed to progressively verify the effects of CN/rGO-mediated macrophage polarization regulation on downstream neurogenesis, angiogenesis, and osteogenic differentiation.

### 2.6 CN/rGO hydrogel promotes sensory neurogenesis through immunomodulation

**Fig. 6:**
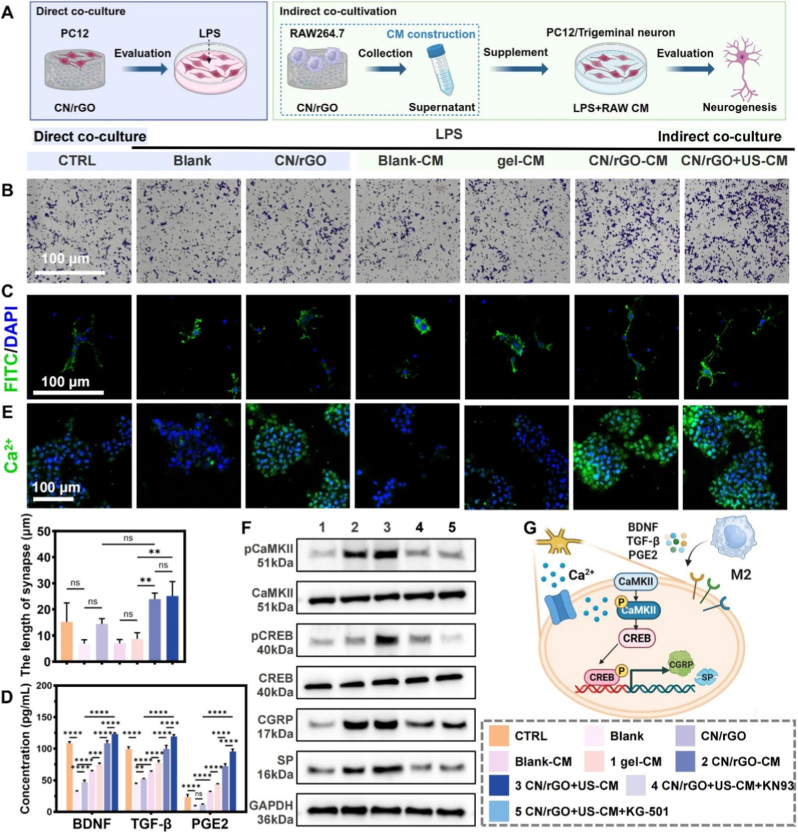
CN/rGO + US promotes sensory neurogenesis through immunomodulation. (A) Schematic illustration of the experimental design using direct culture and conditioned medium (CM) indirect culture systems to evaluate immune–neural interactions. (B) Transwell migration assay showing the effects of different CMs on PC12 cell migration. (C) Immunofluorescence images and quantification of trigeminal ganglion (TG) neuron axonal extension under different CM treatments. (D) ELISA detection of neurotrophic factor concentrations (BDNF, TGF-β, PGE2) secreted by M2 macrophages in different CMs. (E) Ca^2+^ fluorescence imaging of PC12 cells under different CM treatments. (F) Western blot analysis of CGRP, SP, p-CaMKII, CaMKII, p-CREB, and CREB protein expression levels in PC12 cells treated with the CaMKII inhibitor KN-93 or the CREB inhibitor KG-501. (G) Schematic illustration of the “immune–neural” regulatory mechanism mediated by CN/rGO + US through the CaMKII/CREB pathway. Data are means ± SD (n = 3). One-way ANOVA or Two-way ANOVA was followed by Dunnett's test. *P < 0.05, **P < 0.01, ***P < 0.001, ****P < 0.000, ns = not significant.

It has been demonstrated that CN/rGO + US can induce macrophage M2 polarization through inhibition of the JAK-STAT pathway. Bone healing is a temporally coordinated process accompanied by early immune responses and neurovascular regeneration, in which macrophage-secreted neurotrophic factors play a pioneering role in the reconstruction of intraosseous sensory nerves^14^. Therefore, the effects of CN/rGO hydrogel on neurogenesis through modulation of the immune microenvironment were subsequently investigated (Fig. 6A). Cytoskeletal staining (Fig. S13), CCK-8 assay (Fig. S14), and live/dead staining (Fig. S15) results showed that PC12 cell numbers were reduced under LPS stimulation, with some cells exhibiting shrinkage and death, whereas cells cultured in the CN/rGO + US group for 3 days displayed a larger spreading area and significantly improved viability compared with the LPS group. Western blotting results (Fig. S16) demonstrated that CN/rGO + US enhanced the expression of BDNF and Bcl-2, promoting neuronal survival under LPS-induced conditions.

To distinguish between the direct effects of CN/rGO on PC12 cells and the indirect effects mediated through immunomodulation, Transwell experiments and conditioned medium from CN/rGO + US-treated macrophages (CN/rGO + US-CM) were employed to investigate the neurogenic effects. The results showed that PC12 cells in the CN/rGO + US-CM group exhibited the greatest migration distance compared with all other groups (Fig. 6B–S17). Immunofluorescence results revealed that the mean axonal length of trigeminal neurons in this group reached 25.15 ± 7.84 μm, significantly longer than that in all other groups (including the PC12-only group), providing morphological evidence of its capacity to promote neural regeneration (Fig. 6C). These findings suggest that the pro-neurogenic effect of CN/rGO under ultrasound stimulation is primarily attributable to the regulation of macrophage polarization rather than direct material–neuron interactions. Macrophages secrete factors with neuroprotective and neurotrophic properties that modulate sensory nerve activity^14^. ELISA results showed that BDNF, TGF-β, and PGE2 expression levels in the CN/rGO + US-CM group were significantly higher than those in the other groups (Fig. 6D), suggesting that the CN/rGO hydrogel under ultrasonication promotes neurotrophic factor secretion by modulating macrophage phenotype, thereby facilitating neural axon extension and cell migration to drive neural regeneration. This effect is mediated primarily by macrophage paracrine signaling. The direct co-culture and conditioned-medium systems were established to distinguish the direct effect of the material on effector cells from the indirect, macrophage-mediated effect, rather than to compare the two under matched conditions, since their contact time and piezoelectric field coverage differ [28]. The sustained advantage exhibited by the conditioned-medium group therefore indicates that immunomodulation is the principal contributing pathway.

Ca^2+^ fluorescence staining results (Fig. 6E) showed that intracellular calcium ion concentration was significantly decreased in the LPS group compared with the control, whereas intracellular calcium signaling was restored in the CN/rGO + US-CM group. Western blotting results demonstrated that neuropeptide (CGRP, SP) expression in this group was significantly upregulated compared with the LPS group, accompanied by enhanced activation levels of pCaMKII and pCREB (Fig. 6F–S18). When the CaMKII inhibitor KN-93 and the CREB inhibitor KG-501 were individually added to CN/rGO + US-treated RAW-CM, both inhibitors significantly reduced CGRP and SP expression. This demonstrates that CN/rGO + US indirectly activates the neuronal CaMKII/CREB pathway through macrophages, enhancing neuropeptide synthesis and release (Fig. 6G). In summary, these findings reveal a piezoelectric-driven “immune–neural” regulatory mechanism: CN/rGO, upon ultrasound activation, induces macrophage M2 polarization, stimulating the secretion of neurotrophic factors including BDNF, TGF-β, and PGE2, which activate the CaMKII/CREB pathway in sensory neurons, upregulate the expression of key neuropeptides such as CGRP and SP, and ultimately promote neural axon growth and intraosseous sensory reconstruction.

### 2.7 CN/rGO promotes angiogenesis through immunomodulation

**Fig. 7:**
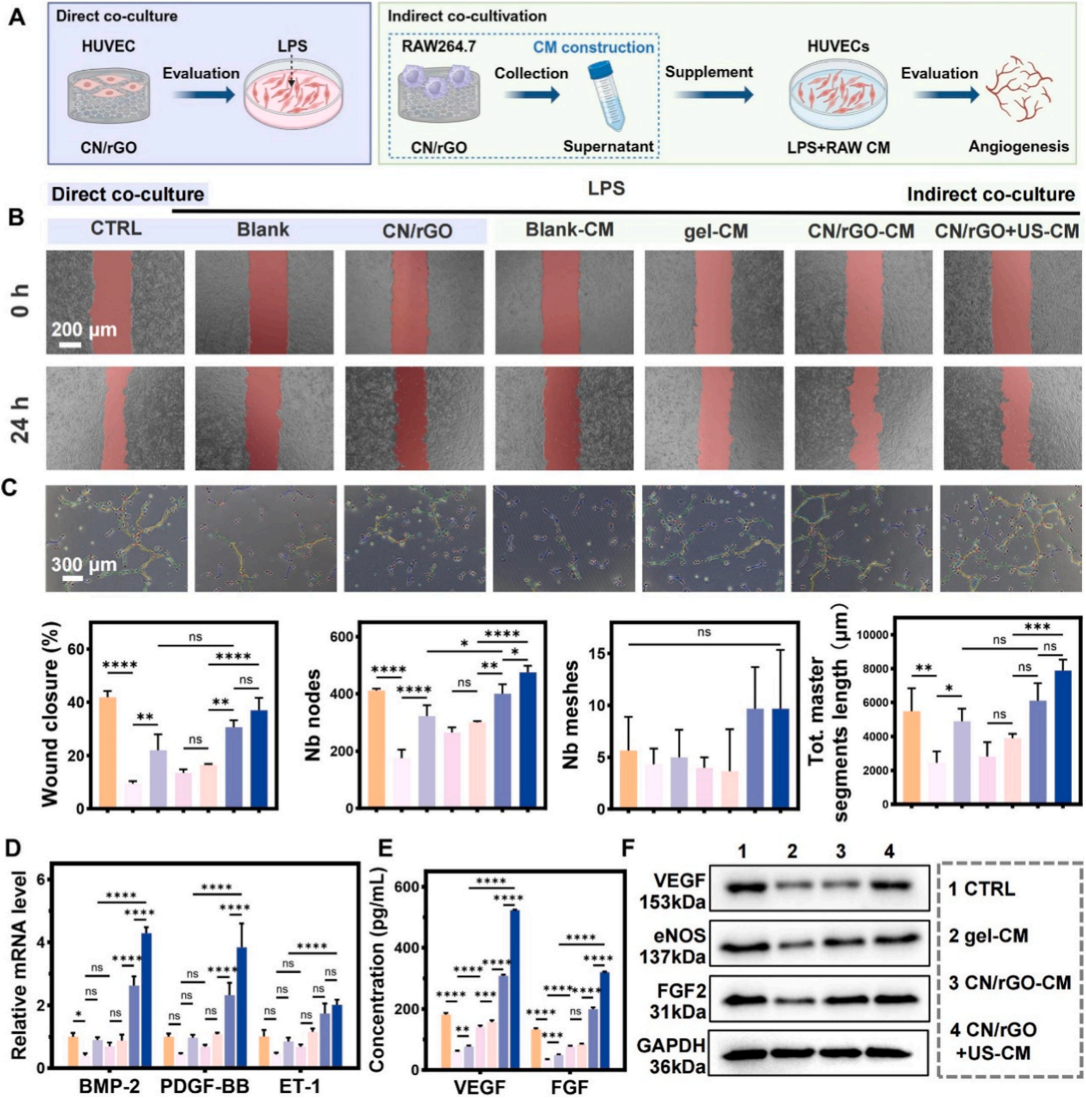
CN/rGO + US promotes angiogenesis through immunomodulation. (A) Schematic illustration of the experimental design using direct culture and CM indirect culture systems to evaluate immune–vascular interactions. (B) Scratch wound healing assay showing HUVEC migration capacity under different treatments. (C) Matrigel tube formation assay showing vascular network morphology. (D) RT-qPCR detection of osteogenic coupling factor (BMP-2, PDGF-BB, ET-1) expression levels in HUVECs under different CM treatments. (E) ELISA detection of pro-angiogenic factor concentrations (VEGF, FGFs, TNF-α, MMP-9) secreted by macrophages in different CMs. (F) Western blot analysis of angiogenesis-related protein expression levels in HUVECs. Data are means ± SD (n = 3). One-way ANOVA or Two-way ANOVA was followed by Dunnett's test. *P < 0.05, **P < 0.01, ***P < 0.001, ****P < 0.000, ns = not significant.

In addition to sensory nerves, the vascular network is also a critical component of the osteogenic microenvironment, delivering oxygen, nutrients, and growth factors to osteocytes to facilitate bone remodeling. To investigate the effects of CN/rGO on angiogenesis, macrophage conditioned medium was used to stimulate human umbilical vein endothelial cells (HUVECs) to simulate the immune–vascular interactive microenvironment (Fig. 7A). Live/dead staining showed that LPS induction significantly inhibited HUVEC growth, with the appearance of dead cells. The CN/rGO group exhibited no appreciable abnormalities in cell proliferation (Fig. S19), indicating good biocompatibility with endothelial cells, which was further confirmed by CCK-8 assay results (Fig. S20). Cytoskeletal staining revealed that HUVECs in the LPS group displayed a rounded and contracted morphology, whereas cells cultured on the CN/rGO hydrogel exhibited elongated cell bodies with pseudopodia formation, resembling the morphology of the control group, indicating that CN/rGO can restore endothelial cell spreading and adhesion capacity under inflammatory conditions (Fig. S21). Scratch assay results showed that cell migration in the LPS group was significantly reduced compared with the control. Both the CN/rGO + US group and the CN/rGO + US-CM group significantly promoted HUVEC migration, although the difference between these two groups was not statistically significant (Fig. 7B). However, in the tube formation assay, the tubular structures in the CN/rGO + US-CM group were significantly superior tubular structures in the CN/rGO + US-CM group were significantly superior to those in the CN/rGO + US direct culture group in terms of total tube length, number of branch points, and number of meshes (Fig. 7C). This indicates that during the more complex process of lumen formation, pro-angiogenic factors such as VEGF and FGFs secreted by M2 macrophages through paracrine signaling play an indispensable regulatory role, and the advantage of the immunomodulatory pathway becomes evident at the functional level. These findings suggest that the pro-angiogenic effect of CN/rGO exerted indirectly through macrophages under ultrasound activation surpasses the direct stimulation of endothelial cells by the material.

During bone remodeling, vascular endothelial cells can secrete pro-osteogenic factors via paracrine signaling to promote the osteogenic differentiation of bone marrow stem cells. PCR results showed that the expression levels of pro-osteogenic factors including BMP-2, PDGF-BB, and ET-1 were highest in endothelial cells of the CN/rGO + US-CM group (Fig. 7D). Under ultrasound application, pro-osteogenic factor expression in this group was significantly higher than in the CN/rGO + US group, confirming that ultrasound-activated CN/rGO promotes endothelial cell secretion of pro-osteogenic factors by acting on macrophages. ELISA results demonstrated that in the indirect culture medium of the CN/rGO + US-CM group, the expression of macrophage-secreted angiogenesis-related factors VEGF and fibroblast growth factors (FGFs) was significantly higher than in the other groups (Fig. 7E and F), suggesting that CN/rGO can promote angiogenesis by enhancing RAW macrophage secretion of angiogenesis-related factors under ultrasound stimulation. In summary, CN/rGO under ultrasound drive modulates macrophage polarization to establish an immune microenvironment favorable for angiogenesis. Combined with the results from the preceding section, these findings confirm that CN/rGO + US can simultaneously induce neurogenesis and angiogenesis through modulation of the immune microenvironment, amplifying neurogenic and angiogenic signals via immunomodulation to remodel the osteogenic microenvironment.

### 2.8 CN/rGO hydrogel promotes osteogenic differentiation of bone marrow mesenchymal stem cells

**Fig. 8:**
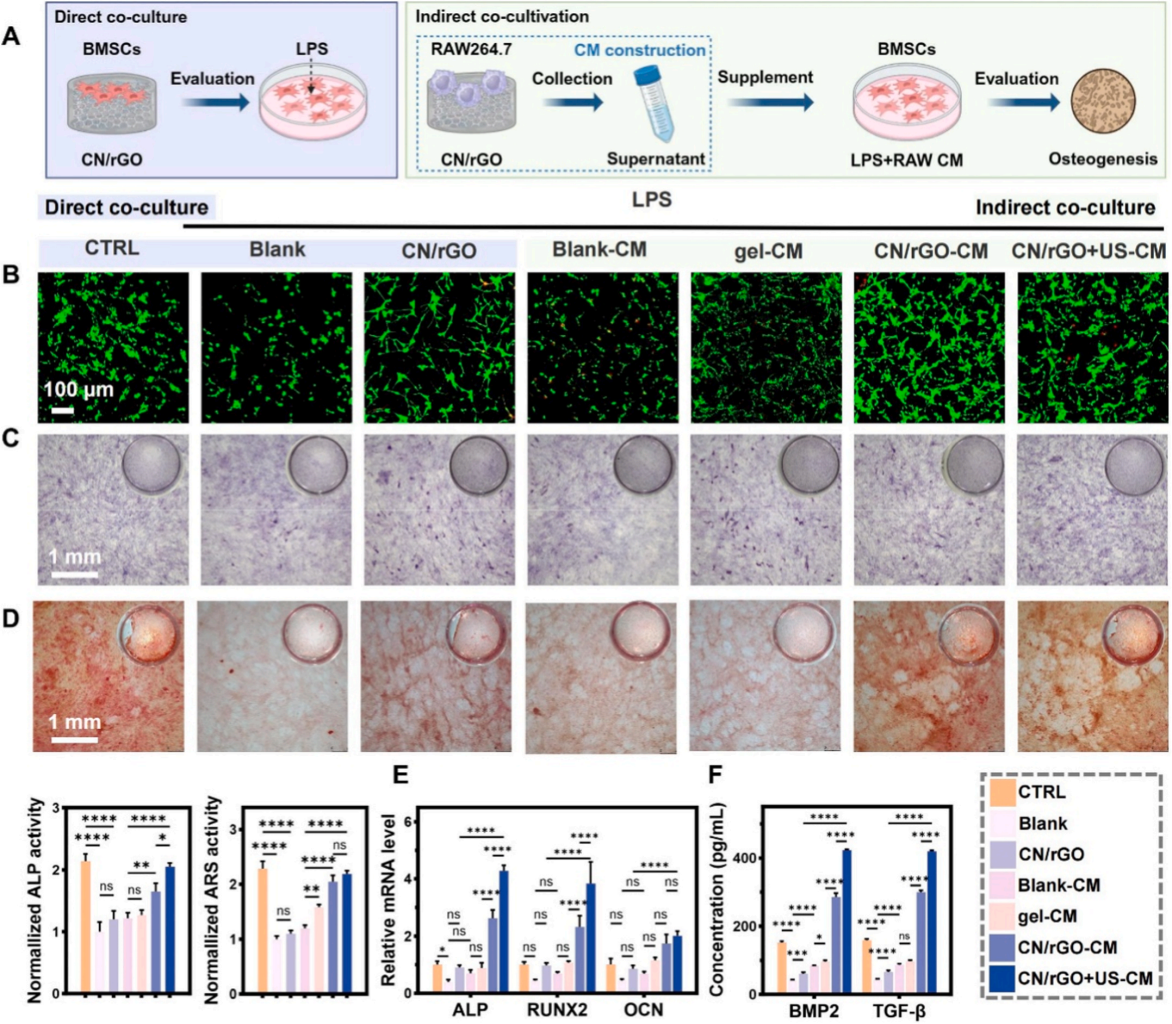
CN/rGO + US promotes osteogenic differentiation of BMSCs through immunomodulation. (A) Schematic illustration of the experimental design using direct culture and CM indirect culture systems to evaluate immune–osteogenic interactions. (B) Live/dead staining of BMSCs cultured under different conditions for 3 days. (C) Alkaline phosphatase (ALP) staining on day 7 and (D) Alizarin Red S (ARS) staining on day 14. (E) RT-qPCR detection of osteogenic marker gene (ALP, OCN, RUNX2) expression. (F) ELISA detection of osteogenic induction factor (BMP-2, TGF-β) concentrations secreted by macrophages in different CMs. Data are means ± SD (n = 3). One-way ANOVA or Two-way ANOVA was followed by Dunnett's test. *P < 0.05, **P < 0.01, ***P < 0.001, ****P < 0.000, ns = not significant. (For interpretation of the references to color in this figure legend, the reader is referred to the Web version of this article.)

Neurogenesis and angiogenesis must ultimately converge upon osteogenic effects to achieve effective osseointegration of implants. Under inflammatory conditions, the osteogenic capacity of bone marrow mesenchymal stem cells (BMSCs) is impaired, and the restoration of their osteogenic function directly determines osseointegration efficacy (Fig. 8A). Live/dead cell staining results (Fig. 8B) showed that LPS significantly inhibited BMSC growth, whereas BMSCs in the CN/rGO + US group exhibited favorable growth, demonstrating the excellent biocompatibility of the material. Alkaline phosphatase (ALP) and Alizarin Red S (ARS) staining results showed that staining intensity was significantly reduced in the LPS group; the CN/rGO + US direct culture group exhibited deeper staining than the LPS group but weaker staining than the CN/rGO + US-CM group. This suggests that CN/rGO can partially restore the osteogenic capacity of BMSCs under ultrasound induction, but its pro-osteogenic effect is primarily mediated through the regulation of macrophages, a trend that became more pronounced by day 14 of culture (Fig. 8C and D). PCR results showed that the transcriptional expression levels of ALP, OCN, and RUNX2 were highest in BMSCs of the CN/rGO + US-CM group (Fig. 8E), confirming that CN/rGO under ultrasound stimulation can indirectly promote osteogenic differentiation and mineralization of BMSCs through the regulation of immune cells. ELISA results demonstrated that the expression levels of osteogenic induction factors BMP-2 and TGF-β were significantly elevated in this group (Fig. 8F). In summary, the ultrasound-activated CN/rGO hydrogel not only directly stimulates BMSC osteogenesis through the piezoelectric field but, more importantly, promotes macrophage M2 polarization and secretion of osteogenic induction factors, thereby facilitating osteogenic differentiation and mineralization of BMSCs under inflammatory conditions in an indirect yet more potent manner.

Collectively, the in vitro results from Sections 2.6–2.8 demonstrate that immunomodulation-mediated indirect effects consistently outperform direct material stimulation across neurogenesis, angiogenesis, and osteogenesis, confirming that CN/rGO + US remodels the osteogenic microenvironment in periodontitis through the cascade of “immunomodulation → neurogenesis/angiogenesis → osteogenic differentiation."

### 2.9 Effects of CN/rGO hydrogel on in vivo implant osseointegration

**Fig. 9:**
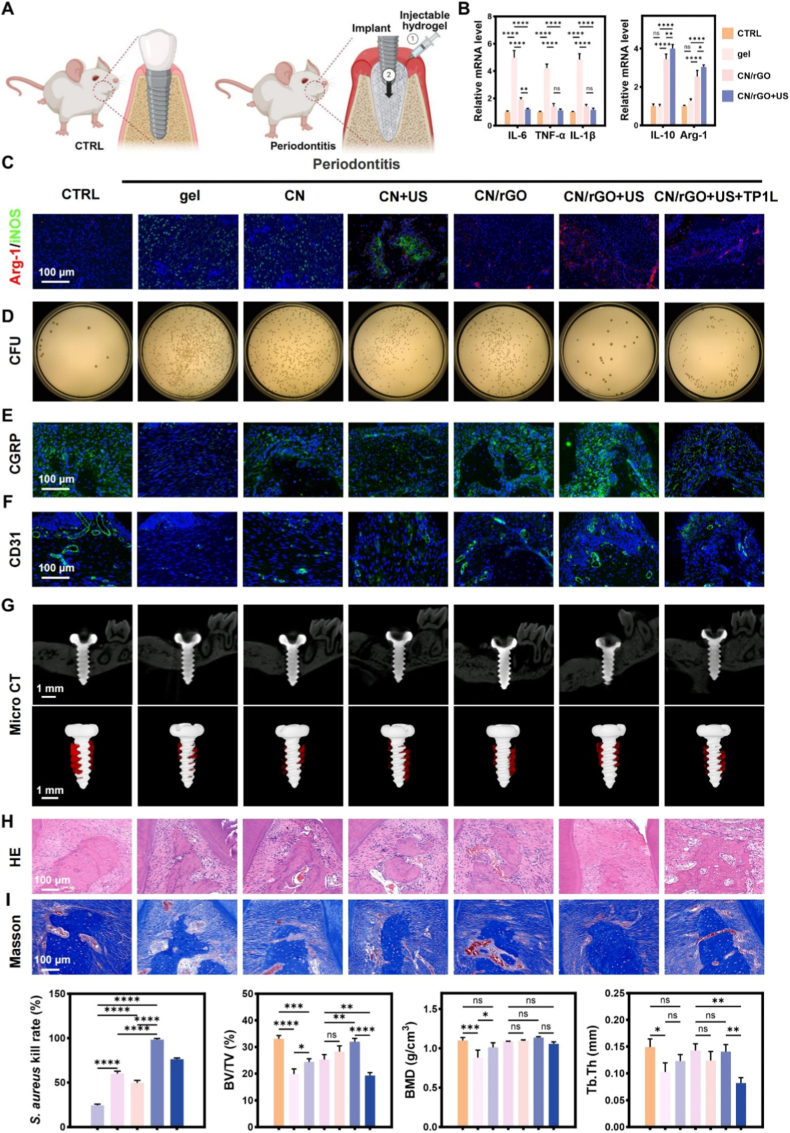
In vivo validation of CN/rGO + US-mediated immunomodulation promoting implant osseointegration in a rat periodontitis model. (A) Schematic illustration of the animal experimental design, including the ultrasound intervention protocol (postoperative days 1–14). (B) RT-qPCR analysis of pro-inflammatory cytokines (IL-6, TNF-α, IL-1β) in peri-implant tissues at 1 week post-implantation. Data are means ± SD (n = 3). Two-way ANOVA was followed by Dunnett's test. *P < 0.05, **P < 0.01, ***P < 0.001, ****P < 0.000, ns = not significant. (C) Immunofluorescence staining of Arg-1 and iNOS at 2 weeks post-implantation, and changes following TP1L intervention. (D) CFU quantification of bacteria on the implant surface at 4 weeks post-implantation. Immunofluorescence staining of (E) CGRP^+^ sensory nerve fibers and (F) CD31^+^ neovessels in the peri-implant region at 4 weeks post-implantation. (G) Representative Micro-CT three-dimensional reconstruction images of peri-implant bone formation at 8 weeks post-implantation. Quantitative analysis of bone volume fraction (BV/TV) across groups (n = 3). (H–I) Hematoxylin and eosin (H&E) and Masson's trichrome staining of peri-implant tissues at 8 weeks post-implantation.

To validate the above in vitro findings at the in vivo level, a rat periodontitis implant model was employed to comprehensively evaluate the therapeutic efficacy and biological mechanisms of the CN/rGO hydrogel across multiple dimensions, including antibacterial activity, immunomodulation, neurovascular regeneration, and osseointegration (Fig. 9A). At placement, the injectable hydrogel filled the marginal jumping gap that invariably remains between an immediately placed implant and the socket wall. It occupied this peri-implant space—where bacteria colonize and bone defects form, and where antibacterial and osteogenic regulation are most needed—without competing with the implant for space [33]. Micro-CT and histological analyses showed that CN/rGO + US markedly improved peri-implant bone formation and osseointegration, restoring these parameters to levels approaching those of the healthy, non-inflamed control group. The in vivo ultrasound intervention protocol was as follows: from postoperative days 1 to 14, ultrasound stimulation was administered once daily (1.0 MHz, 1.5 W cm^−2^, 50% duty cycle, 10 min per session); ultrasound intervention was discontinued from day 15 onward, and observation continued until week 8. This 2-week window corresponded to both the hydrogel's effective piezoelectric period (Section 2.2) and the M1-to-M2 polarization timeline (Section 2.4).

The regulatory effects of the CN/rGO hydrogel on the peri-implant inflammatory microenvironment were first assessed. At 1 week post-implantation, PCR analysis of periodontal tissues showed that the expression of inflammation-associated genes IL-6, TNF-α, and IL-1β was significantly elevated in the periodontitis group compared with the control group, whereas these inflammatory factors were suppressed in the CN/rGO group, with expression levels approaching those of the control group (Fig. 9B). Immunofluorescence staining demonstrated that the CN/rGO + US group successfully induced macrophage M2 polarization within the periodontitis microenvironment, as evidenced by significantly elevated Arg-1 expression and downregulated iNOS expression in local tissues at 2 weeks post-implantation (Fig. 9C). Following local injection of the JAK1 pathway activator TP1L, the number of iNOS-positive cells increased again, with the local tissue reverting to a pronounced pro-inflammatory state, confirming that in vivo immunomodulation likewise depends on the inhibition of the JAK-STAT pathway.

At 4 weeks, the bacterial load on the implant surface in the CN/rGO + US group was significantly reduced (Fig. 9D), which is associated with the antibacterial properties of ultrasound-catalyzed ROS generation in this group, validating the effectiveness of the “short-term effect” in vivo. Concurrent immunofluorescence showed that the density of CGRP^+^ sensory nerve fibers and CD31^+^ neovessels in the peri-implant region of the CN/rGO + US group reached the highest levels (Fig. 9E and F). However, in the TP1L intervention group, the degree of innervation and vascularization was significantly diminished, further confirming that CN/rGO induces neurovascular regeneration by suppressing the JAK-STAT pathway and remodeling the immune microenvironment.

At 8 weeks post-implantation, micro-computed tomography (Micro-CT) three-dimensional reconstruction and quantitative analysis of peri-implant bone tissue demonstrated that the CN/rGO + US group exhibited the most favorable osteogenic efficacy, with a bone volume fraction (BV/TV) significantly higher than that of the hydrogel-alone group (Fig. 9G). Following local administration of TP1L, the pro-osteogenic effect induced by CN/rGO was markedly attenuated, with bone volume decreasing to levels approaching those of the inflammation group, indicating that inhibition of the JAK-STAT pathway is a critical step for CN/rGO to exert its pro-osteogenic effects. H&E and Masson's trichrome staining further confirmed that the CN/rGO + US group exhibited the greatest collagen deposition and new bone formation (Fig. 9H and I).

In summary, the in vivo experiments systematically validated the “short-term effect–long-term effect” synergistic strategy of the CN/rGO hydrogel from a temporal perspective: during the ultrasound intervention period (first 2 weeks), the piezoelectric field drove ROS-mediated antibacterial action and initiated macrophage M1-to-M2 polarization reprogramming; after cessation of ultrasound (weeks 2–4), the established M2-type immune microenvironment continuously drove neurovascular regeneration through paracrine signaling; in the long term (weeks 4–8), neurovascular–osteogenic coupling effects promoted bone tissue remodeling, achieving efficient osseointegration. H&E staining at 8 weeks revealed no histological damage to major organs, confirming the biosafety of the material. The ultrasound-driven CN/rGO hydrogel can reverse the chronic inflammatory microenvironment of periodontitis, promote integrated “nerve–vessel–bone” regeneration, and ultimately achieve implant osseointegration under pathological conditions. It should be noted that the 1.0 MHz ultrasound used in this study readily penetrates soft tissue, and within the oral cavity the hydrogel resides at a superficial peri-implant site beneath a thin layer of mucosa; clinical dental equipment can provide sufficient energy, and the moist environment further facilitates acoustic coupling. However, deeper jawbone attenuates the signal, so both the dosage and dedicated intraoral transducers require further optimization [34]. The model also differs from the human disease: rats underwent induced periodontitis with immediate implantation and a defined flora over several weeks, whereas human periodontitis is a chronic, polymicrobial infection that develops over many years and is characterized by different bone turnover, occlusal loading, and immune responses [35]. The present conclusions therefore remain to be confirmed in large-animal studies and clinical research.

## 3 Conclusions

In this study, a piezoelectric conductive hydrogel loaded with CN/rGO two-dimensional nano-heterostructures was constructed to achieve synergistic “early antibacterial–long-term immunomodulatory” effects for implant osseointegration under pathological conditions. Through efficient carrier separation and ultrasound-mediated ROS generation, the hydrogel eradicates pathogenic bacteria and biofilms via a “piezoelectric–ROS” cascade reaction while precisely regulating macrophage polarization — inducing M1 in the early phase and M2 in the later phase — to promote neurogenesis, angiogenesis, and osteogenic differentiation through paracrine effects. Validation in a rat periodontitis implant model demonstrated that the ultrasound-driven CN/rGO hydrogel effectively suppressed local inflammation, enhanced peri-implant neurogenesis and angiogenesis, promoted new bone formation and osseointegration. Mechanistic studies established that inhibition of the JAK-STAT signaling pathway is the core mechanism underlying its regulation of macrophage polarization and its immunomodulatory and pro-osteogenic effects. In conclusion, the CN/rGO piezoelectric conductive hydrogel constructed in this study achieves spatiotemporal synergy between early antibacterial action and long-term tissue regeneration, offering a feasible new approach for improving the success rate of dental implant restoration in periodontitis patients.

## 4 Experimental methods

Detailed information is provided in the Supplementary Materials.

## CRediT authorship contribution statement

**Dan Li:** Data curation, Methodology, Writing – original draft, Writing – review & editing. **Zhen Ai:** Methodology, Writing – review & editing. **Jinyang Lv:** Writing – review & editing. **Yijun Zheng:** Writing – review & editing. **Chao Zhang:** Conceptualization, Data curation, Funding acquisition, Methodology, Project administration, Resources, Software, Supervision, Writing – original draft, Writing – review & editing.

## Declaration of competing interest

The authors declare that they have no known competing financial interests or personal relationships that could have appeared to influence the work reported in this paper.

## Data Availability

Data will be made available on request.
