# Supplementary material for: Piezoelectric g-C3N4/rGO heterojunction remodels the periodontitis immune microenvironment to promote neurovascular–bone coupled osseointegration
Source: Mater Today Bio. 2026 Jul 27;39:103498. doi: 10.1016/j.mtbio.2026.103498 (PMC13450610; doi:10.1016/j.mtbio.2026.103498)
Supplement: Supplementary file 1 [file mmc1.docx]

**Supplementary Materials**

**Piezoelectric g-C₃N₄/rGO Heterojunction Remodels the Periodontitis Immune Microenvironment to Promote Neurovascular–Bone Coupled Osseointegration**

# 1. Materials and Methods

## 1.1. Materials

Graphitic carbon nitride (g-C₃N₄) and graphene oxide (GO) were purchased from Jiangsu XFNANO Materials Tech Co., Ltd. (Jiangsu, China). *L*-Ascorbic acid powder was obtained from Sigma-Aldrich (St. Louis, MO, USA). All chemicals were of analytical grade and used without further purification. Dulbecco’s modified Eagle’s medium (DMEM), fetal bovine serum (FBS), penicillin/streptomycin (100 units/mL), and EDTA/trypsin (0.25%) were purchased from Gibco® Life Technologies (Grand Island, NY, USA). The Cell Counting Kit-8 (CCK-8), BCIP/NBT alkaline phosphatase staining kit, and lysis buffer were provided by Beyotime Biotechnology Co., Ltd. (Shanghai, China). BMSC osteogenic differentiation medium and alizarin red dye were purchased from Cyagen Biosciences (Jiangsu, China). Phosphate-buffered saline (PBS), 4% paraformaldehyde, and Triton X-100 were obtained from Solarbio (Beijing, China). TRIzol reagent was provided by Invitrogen Life Technology (Carlsbad, CA, USA), and the SYBR Green Pro Taq HS Premixed qPCR Kit II was supplied by Hunan Accurate Bioengineering Co., Ltd. The BCA protein assay kit was purchased from Bio-Rad (Hercules, CA, USA), polyvinylidene fluoride (PVDF) membranes from Millipore (Billerica, MA, USA), and the enhanced chemiluminescence (ECL) kit from Amersham Biosciences (Piscataway, NJ, USA). Primary antibodies were purchased from Abcam (Cambridge, UK). Secondary antibodies, fluorescent secondary antibodies, fluorescent dyes, the JAK1-specific activator TP1L, and the JAK1-specific inhibitor upadacitinib were purchased from Sigma-Aldrich (St. Louis, MO, USA). Masson’s trichrome staining reagent was purchased from Thermo Fisher Scientific (Shanghai, China).

Primary antibodies used in this study included anti-Arg-1 and anti-iNOS (Abcam); anti-pCaMKII, anti-CaMKII, anti-pCREB, anti-CREB, anti-CGRP, anti-SP, anti-phospho-JAK1 (p-JAK1), total JAK1, anti-phospho-STAT1 (p-STAT1), total STAT1, and anti-GAPDH (Proteintech, Wuhan, China). ELISA kits for BDNF, TGF-β, PGE2, VEGF, FGF, and BMP-2 were purchased from Thermo Fisher Scientific. Hematoxylin and eosin staining kits were purchased from Servicebio Technology Co., Ltd.

## 1.2. Nanomaterial synthesis and characterization

GO nanosheets (0.05 g) were dispersed in 50 mL of deionized water by ultrasonication for 4 h to prepare a 1.0 mg/mL GO suspension. L-Ascorbic acid powder (50 mg) was added to the GO solution, and the reduction reaction was carried out in a water bath at 80 °C under magnetic stirring at 500 rpm for 3 h. The product was washed with deionized water and the pH was adjusted to approximately 7–8. The rGO suspension was freeze-dried for 24 h to obtain the rGO sample. For protonation, g-C₃N₄ (0.05 g) was dispersed in aqueous HCl solution (0.5 M, 10 mL) and ultrasonicated in a flask for 1 h. After washing twice each with deionized water and ethanol, the product was freeze-dried to obtain protonated g-C₃N₄.

To optimize the CN/rGO heterojunction composition, heterojunctions with different rGO contents (10, 15, and 20 wt%) were prepared (Fig. S1) and screened based on two criteria: ROS generation capacity (MB degradation rate) and electrical conductivity (EIS). Results indicated that 15 wt% rGO yielded the best overall performance, and this ratio was adopted for all subsequent experiments. Specifically, rGO (at a mass fraction of 15% relative to g-C₃N₄) and protonated g-C₃N₄ were combined in deionized water in a flask and stirred magnetically at 80 °C for 3 h. The resulting mixture was washed multiple times with deionized water and ethanol and then freeze-dried for 24 h to obtain g-C₃N₄/rGO (CN/rGO).

The morphology and dimensions of the nanomaterials were characterized by high-resolution transmission electron microscopy (HRTEM; Tecnai G2 F20 S-TWIN, USA). Functional groups and structural features were analyzed by FTIR and Raman spectroscopy (excitation wavelength: 513 nm; Renishaw, UK). The crystal structure of CN/rGO was characterized by X-ray diffraction (XRD). Elemental composition and chemical bonding states were identified by X-ray photoelectron spectroscopy (XPS; Thermo Fisher Scientific K-Alpha), with the C 1s peak at 284.6 eV used for calibration.

## 1.3. First-principles calculations

First-principles calculations were carried out on the basis of periodic density functional theory (DFT) using a generalized gradient approximation within the Perdew–Burke–Ernzerhof exchange–correlation functional. Geometry optimization was performed prior to energy calculations. Plane-wave basis sets with an energy cutoff of 500 eV were employed, and a Gamma-centered k-point mesh of 3 × 3 × 1 was used. Convergence tolerances for geometry optimization were set at 1.0 × 10⁻⁶ eV/atom for total energy and 0.05 eV/Å for force, respectively. To avoid spurious interactions between periodic images, a vacuum gap of 15 Å was applied in the periodically repeated slabs. Monolayer models of g-C₃N₄ and rGO were constructed, and the CN/rGO heterojunction model was optimized to evaluate band alignment, charge-density difference (Δρ), and the direction of interfacial charge transfer.

## 1.4. Electrochemical and piezoelectric measurements

Electrochemical impedance spectroscopy (EIS) and cyclic voltammetry (CV) were performed using an electrochemical workstation (CHI 760E) in 0.1 M Na₂SO₄ electrolyte via a standard three-electrode system with a Pt counter electrode and an Ag/AgCl reference electrode. Piezoelectric response was evaluated by piezoresponse force microscopy (PFM; Bruker Dimension Icon) using a conductive SCM-PIT-V2 probe. The local piezoelectric coefficient (d₃₃) was calculated from the amplitude–voltage butterfly loop curves according to the equation: d₃₃ = Δz/V*ac*, where Δz is the vertical displacement and V*ac* is the applied AC voltage.

## 1.5. Evaluation of piezo-catalytic performance

The piezo-catalytic generation of reactive oxygen species (ROS) was evaluated by monitoring the degradation of methylene blue (MB). For concentration screening, CN/rGO heterojunction dispersions at different concentrations (0.025, 0.05, 0.1, 0.2, and 0.5 mg/mL) were added to an MB solution (10 mg/L) and subjected to ultrasound irradiation (1.0 MHz, 1.5 W cm⁻², 50% duty cycle, 5 min). The ·OH generation capacity as a function of concentration was assessed by measuring absorbance at 664 nm (Fig. S3). Results showed that ·OH generation plateaued above 0.1 mg/mL; this concentration was therefore used for all subsequent experiments. For time-dependent measurements, a 0.1 mg/mL catalyst dispersion was added to the MB solution under ultrasound irradiation, and the supernatant absorbance at 664 nm was measured at regular intervals. Electron paramagnetic resonance (EPR) spectroscopy was performed at 298 K using a Bruker A300-10/12 spectrometer (Bruker, Billerica, MA, USA).

## 1.6. Fabrication and characterization of the CN/rGO hydrogel

4-(Bromoethyl)phenylboronic acid (1 g, 4.6 mmol) and N,N,N′,N′-tetramethyl-1,3-propanediamine (0.2 g, 1.5 mmol) were dissolved in N,N-dimethylformamide (40 mL) and stirred at 60 °C for 24 h. Tetrahydrofuran (THF, 100 mL) was then added to the supernatant to precipitate a white solid, which was resuspended in 20 mL THF, centrifuged, and freeze-dried to yield tsPBA as a white solid. Based on our prior work, a formulation of 3 wt% tsPBA and 6 wt% PVA was adopted to satisfy the requirements for injectability and gelation. The 3 wt% tsPBA aqueous solution was mixed with the 6 wt% PVA aqueous solution, and CN/rGO nanoparticles (0.5 wt% relative to total hydrogel mass) were added with stirring for 30 min to obtain the CN/rGO hydrogel.

To determine the optimal CN/rGO loading concentration within the hydrogel, formulations with different CN/rGO contents (0.25, 0.5, and 1.0 wt%) were prepared and evaluated for ROS generation capacity (MB degradation rate) and cytocompatibility (CCK-8). Balancing functional efficacy with biosafety, 0.5 wt% was selected as the optimal loading concentration and used in all subsequent experiments.

Microstructure and elemental composition were characterized by scanning electron microscopy (SEM) and energy-dispersive X-ray spectroscopy (EDS). Rheological behavior, including storage modulus (G′), loss modulus (G′′), and viscosity, was characterized using a rotational rheometer (NETZSCH Kinexus Lab+, Germany). Frequency-sweep parameters: 0.1–10 Hz, 37 °C, angular velocity of 5 rad/s. Strain-amplitude sweep parameters: strain from 0.01% to 100%, 37 °C, 10 Hz. Viscosity was measured over a shear-rate range of 0.1–10 s⁻¹. Injectability was qualitatively assessed by extruding the hydrogel through a syringe fitted with a 0.9 × 38 mm needle.

**Swelling and degradation behavior:** The swelling ratio was determined by placing pre-weighed dry hydrogel discs (W*d*) in PBS (pH 7.4) at 37 °C. At predetermined time points, hydrogels were removed, surface-blotted, and weighed (W*s*). The swelling ratio was calculated as (W*s* – W*d*)/W*d* × 100%. In vitro degradation was assessed by monitoring mass loss during a 15-day incubation in PBS. Residual mass was recorded at intervals, and mass-loss percentages were calculated relative to the initial weight.

**Assessment of piezo-catalytic function during degradation:** CN/rGO hydrogels were immersed in PBS or lysozyme-containing PBS (1 mg/mL, 37 ℃) to simulate the in vivo degradation environment. Among them, PBS containing lysozyme is intended to provide an in vivo simulated medium with enzymatic activity [1]. At different degradation time points, samples were collected and the ultrasound-mediated (1.0 MHz, 1.5 W cm⁻², 50% duty cycle, 5 min) ·OH catalytic generation capacity was assessed via MB oxidation (Fig. S7). Additionally, piezoelectric current output under ultrasound was directly measured on hydrogel samples from days 1 and 14 (Fig. S7b) using an electrometer (Tektronix 6514) to record short-circuit current.

**Electrochemical and piezoelectric output characterization:** Electrochemical impedance and CV curves of the hydrogel were measured using an electrochemical workstation (CHI 760E) in a two-electrode configuration, with the hydrogel itself serving as the working electrode. Electrical conductivity was qualitatively verified by integrating the hydrogel into a simple circuit to power a light-emitting diode (LED). For piezoelectric performance measurements, equilibrium-swollen hydrogels were placed in a compression apparatus at room temperature, and the voltage and current output generated by the CN/rGO hydrogel under ultrasound irradiation at different intensities (1.0 MHz, 0.5–2.0 W cm⁻², 50% duty cycle) were recorded using an electrometer (Tektronix 6514, China).

## 1.7. In vitro antibacterial performance

The antibacterial efficacy of the hydrogel was evaluated against three major pathogens associated with periodontitis and peri-implantitis: *Porphyromonas gingivalis* (ATCC 33277), *Fusobacterium nucleatum* (ATCC 25586), and *Staphylococcus aureus* (ATCC 25923). Anaerobic bacteria (*P. gingivalis* and *F. nucleatum*) were cultured in brain–heart infusion (BHI) broth supplemented with hemin and vitamin K under anaerobic conditions (80% N₂, 10% H₂, 10% CO₂) at 37 °C. *S. aureus* was cultured in fresh Luria–Bertani (LB) broth at 37 °C. Antibacterial activity was quantified by colony-forming unit (CFU) counting. Briefly, bacterial suspensions (∼10⁸ CFU/mL) were co-incubated with the corresponding hydrogels in 24-well plates for 48 h. For the ultrasound group, ultrasound irradiation (1.0 MHz, 1.5 W cm⁻², 50% duty cycle, 5 min) was applied using a therapeutic ultrasound device. After incubation, the mixtures were serially diluted and plated on agar plates for CFU counting after 24 h of culture. Bacterial morphology after different treatments was observed by SEM: samples were fixed in 2.5% glutaraldehyde at 4 °C overnight, followed by graded ethanol dehydration (30%, 50%, 70%, 80%, 90%, 100%). After critical-point drying and gold sputter-coating, samples were imaged by SEM.

**Biofilm formation and anti-biofilm efficacy:** To form multi-species biofilms, *P. gingivalis*, *F. nucleatum*, and *S. aureus* were co-inoculated onto sterile coverslips in 12-well plates and cultured for 5 days under their respective conditions. Mature biofilms were then treated with hydrogels with or without ultrasound stimulation. Biofilm viability and thickness were assessed using the MycoLight™ Rapid Fluorescence Gram-Positive Bacteria Staining Kit (AAT Bioquest Inc.). Total biofilm biomass was quantified by crystal violet (CV) staining: after treatment, biofilms were fixed with methanol, stained with 0.1% crystal violet for 15 min, washed thoroughly, and dissolved in 33% acetic acid. Absorbance of the dissolved solution was measured at 600 nm. Images were analyzed using ImageJ.

## 1.8. Biocompatibility

For in vitro experiments, the murine macrophage cell line RAW 264.7 was cultured in DMEM supplemented with 10% FBS and 1% penicillin–streptomycin. The rat pheochromocytoma cell line (PC12) and primary trigeminal ganglion neurons were used as in vitro models for neurogenesis assessment. PC12 cells were maintained in RPMI-1640 medium supplemented with 10% horse serum, 5% FBS, and 1% penicillin–streptomycin. Primary trigeminal neurons were isolated and cultured in Neurobasal medium supplemented with B-27 and 2 mM glutamine. Human umbilical vein endothelial cells (HUVECs) were cultured in endothelial cell medium (ECM) supplemented with 10% FBS, 1% endothelial cell growth supplement (ECGS), and 1% penicillin–streptomycin. Bone marrow-derived mesenchymal stem cells (BMSCs) were isolated from rat femurs and tibiae and cultured in α-minimum essential medium (α-MEM) supplemented with 10% FBS, 1% penicillin–streptomycin, and 1% L-glutamine. All cells were maintained at 37 °C in a humidified atmosphere containing 5% CO₂.

**Establishment of the inflammatory model:** To simulate the periodontitis microenvironment, all in vitro inflammation models employed lipopolysaccharide (LPS, 100 ng/mL) as the sole stimulus. The inflammatory stimulation conditions for all in vitro experiments throughout this study (immunomodulation, conditioned medium, and direct culture) were uniformly LPS.

**Ultrasound stimulation:** Cultures were subjected to daily ultrasound irradiation (1.0 MHz, 1.0 W cm⁻², 50% duty cycle, 10 min per session).

After 3 days of culture, cell viability was assessed by Calcein-AM/PI staining and visualized by confocal laser scanning microscopy (CLSM; FV1000, Olympus, Japan). The percentage of live cells was analyzed using ImageJ software (NIH, Bethesda, MD, USA). Different hydrogel samples were sterilized under UV irradiation for 30 min, placed in 12-well plates, and immersed in 2 mL DMEM at 37 °C for 24 h to obtain extracts. CCK-8 assays were performed on day 3 of co-culture with the respective extracts. After 1 day of culture, cells were fixed with 4% paraformaldehyde, the cytoskeleton was stained with FITC-phalloidin (100 nM), and nuclei were counterstained with DAPI. Cytoskeletal morphology was observed by CLSM.

## 1.9. Phenotypic assessment of macrophage polarization

RAW 264.7 macrophages were stimulated with LPS (100 ng/mL) to induce M1-type polarization as the inflammatory model baseline. IL-4 (40 ng/mL; R&D Systems, Rahway, NJ, USA) was used to polarize macrophages toward the M2 phenotype as a positive control. RAW 264.7 cells were fixed in 4% (w/v) paraformaldehyde, blocked with 1% (w/v) bovine serum albumin (BSA), and stained with primary antibodies against the M1 marker iNOS and the M2 marker Arg-1, followed by incubation with the corresponding fluorescence-labeled secondary antibodies. Nuclei were counterstained with DAPI. Fluorescence images were acquired by CLSM. Immunofluorescence was performed at days 3 and 7 to evaluate the temporal dynamics of macrophage M1→M2 polarization. To quantify the proportion of M2-type macrophages, cells were harvested, stained with fluorescence-conjugated anti-CD86 and anti-CD206 antibodies, and analyzed by flow cytometry. Appropriate isotype controls were used for gating.

After 48 h of treatment with different hydrogel extracts, macrophages were assessed for mRNA expression levels of M1-associated genes (TNF-α, IL-6, IL-1β) and M2-associated genes (Arg-1, IL-10) as described in Section 1.16.

## 1.10. Transcriptome sequencing

For transcriptome sequencing, macrophages were stimulated with LPS (100 ng/mL) to obtain a more robust M1 polarization baseline and to enhance pathway detection sensitivity. All functional validation experiments (western blot, immunofluorescence) were performed under LPS-only stimulation, consistent with the overall in vitro experimental system.

RAW 264.7 macrophages were cultured under different conditions for 24 h, after which total RNA was extracted using TRIzol reagent (Invitrogen, CA, USA). RNA purity and concentration were determined using a NanoDrop 2000 spectrophotometer (Thermo Scientific, USA). RNA sequencing libraries were sequenced on a NovaSeq X Plus platform. Transcriptome sequencing and analysis were performed by Majorbio Bio-Pharm Technology Co., Ltd. (Shanghai, China).

## 1.11. Functional validation of the JAK–STAT signaling pathway

Based on transcriptomic results indicating significant involvement of the JAK–STAT pathway, functional experiments were conducted. To specifically investigate the role of JAK1, cells were pretreated 1 h prior to hydrogel and ultrasound treatment with the JAK1-specific activator TP1L (1 μM) or the JAK1-specific inhibitor upadacitinib (100 nM). Western blot was performed to detect protein expression levels of phospho-JAK1 (p-JAK1), total JAK1, phospho-STAT1 (p-STAT1), and total STAT1. To assess the functional impact of JAK–STAT pathway modulation on macrophage polarization, cells from different treatment groups (including TP1L- or upadacitinib-treated groups) were collected, stained with fluorescence-labeled antibodies targeting the M2 marker CD206 and the M1 marker CD86, and analyzed by immunofluorescence.

## 1.12. Construction of direct and indirect culture systems

**Direct co-culture system:** PC12 cells, HUVECs, or BMSCs were directly seeded onto the hydrogel or cultured with hydrogel extracts. Groups included: CTRL, effector cells without LPS stimulation; LPS, effector cells stimulated with LPS (100 ng/mL); CN/rGO, LPS-stimulated effector cells cultured on CN/rGO hydrogel; CN/rGO + US, same as the CN/rGO group but with daily ultrasound stimulation (parameters as described above).

**Indirect co-culture system (conditioned medium assays):** RAW 264.7 macrophages were first treated under different conditions to generate conditioned medium (CM). For CM preparation, macrophages were stimulated uniformly with LPS (100 ng/mL), used only during the initial M1 induction phase; before CM was collected, the macrophages were washed with PBS and the medium was replaced with fresh, LPS-free medium for conditioning. CM preparation groups were as follows: Blank-CM, from LPS-stimulated macrophages; Gel-CM, from LPS-stimulated macrophages co-cultured with blank hydrogel; CN/rGO-CM, from LPS-stimulated macrophages cultured in CN/rGO hydrogel; CN/rGO + US-CM, from LPS-stimulated macrophages cultured in CN/rGO hydrogel with ultrasound (US) treatment. Collected CM was subsequently used to treat effector cells.

## 1.13. Assessment of neuronal regeneration

**Migration assay:** PC12 cells were seeded in the upper chamber of Transwell inserts, with different conditioned media added to the lower chamber. After 24 h of incubation, cells that had migrated to the lower surface of the membrane were fixed, stained with crystal violet, and counted.

**Neurite outgrowth analysis:** Trigeminal neurons were cultured with extracts or different conditioned media and immunostained with FITC. Fluorescence images were acquired by confocal microscopy, and the average neurite length per neuron was quantified using ImageJ.

**Neural activity:** mRNA expression levels of neurogenesis markers—synaptophysin (SYN), postsynaptic density protein 95 (PSD95), and growth-associated protein 43 (GAP43)—were assessed by qPCR, with GAPDH as the internal reference gene. To validate the involvement of the CaMKII/CREB pathway, the specific inhibitors KN-93 (CaMKII inhibitor, 10 μM) and KG-501 (CREB inhibitor, 20 μM) were added to the CN/rGO + US-CM prior to neuronal treatment. Western blot was performed to detect expression levels of BDNF, Bcl-2, CGRP, SP, phospho-CaMKII (p-CaMKII), and phospho-CREB (p-CREB), as described in Section 1.17. Concentrations of secreted BDNF, TGF-β, and PGE2 in the culture medium were quantified using commercial ELISA kits according to the manufacturer’s instructions.

**Intracellular calcium imaging:** PC12 cells were co-incubated with the fluorescent calcium indicator Fluo-4 AM and subsequently exposed to different conditioned media. Intracellular Ca²⁺ flux was monitored and quantified over a defined time period using a fluorescence microscope or fluorescence microplate reader.

## 1.14. Assessment of angiogenic capacity

**Scratch wound healing assay:** HUVECs were seeded in 12-well plates to form a monolayer, and a scratch wound was created using a sterile pipette tip. Cells were washed and incubated with different conditioned media. Images of the wound area were captured at 0 and 24 h. Wound closure percentages were calculated using ImageJ.

**Tube formation assay:** Matrigel was polymerized in 96-well plates at 37 °C for 30 min, after which HUVECs were seeded onto the Matrigel surface and cultured with different conditioned media. After 6 h of incubation, tubular structures were imaged under an inverted microscope. Total tube length, number of branch points, and number of meshes per field were quantified using the ImageJ Angiogenesis Analyzer plugin. At least three independent fields were analyzed per group.

RT-qPCR was performed to detect the expression levels of osteogenic/angiogenic coupling factors (BMP-2, PDGF-BB, ET-1) and their inhibitor Noggin in HUVECs treated with different culture supernatants. Concentrations of key angiogenic factors (VEGF, FGFs, TNF-α, MMP-9) in macrophage culture supernatants were quantified using commercial ELISA kits.

## 1.15. Assessment of osteogenic differentiation

To induce osteogenic differentiation, the culture medium was replaced at the start of the experiment with osteogenic differentiation medium containing 10 mM β-glycerophosphate, 50 μg/mL ascorbic acid, and 100 nM dexamethasone. BMSCs in the lower chamber were stained with an ALP staining kit (Beyotime Biotechnology, China) on day 7 and an alizarin red S (ARS) staining kit (Cyagen Biotechnology Co., Ltd., China) on day 14. Medium was changed every 2 days. Cells were fixed with 4% paraformaldehyde and stained according to the manufacturer’s instructions. After removing the staining solution and rinsing with PBS, images were captured and quantified using ImageJ. On day 14, fixed cells were stained with a primary antibody against osteocalcin (OCN), followed by a fluorescence-labeled secondary antibody. Nuclei were counterstained with DAPI, and fluorescence images were acquired by confocal microscopy. RT-qPCR was performed to detect mRNA expression of key osteogenic genes: Alpl (ALP), Bglap (OCN), and Runx2. Concentrations of osteoinductive factors (BMP-2, TGF-β) secreted in macrophage culture supernatants (e.g., CN/rGO + US-CM) were quantified using commercial ELISA kits.

## 1.16. Quantitative real-time reverse transcription PCR (qRT-PCR)

Effector cells co-cultured with conditioned medium were harvested using TRIzol (Invitrogen, Waltham, MA, USA). Extracted RNA was reverse-transcribed into cDNA using a reverse transcription system (Toyobo, Osaka, Japan). Gene expression levels were analyzed using the QuantiTect SYBR Green Kit (Roche, Basel, Switzerland) with cDNA as template on an Applied Biosystems QuantStudio 3 thermal cycler. Primer sequences used for qPCR are listed in Table S1.

## 1.17. Western blot analysis

Cellular proteins were extracted using RIPA lysis buffer (Santa Cruz, USA) and quantified using the BCA protein assay kit (Thermo Scientific, USA). Protein samples were separated by sodium dodecyl sulfate–polyacrylamide gel electrophoresis (SDS-PAGE) and transferred to PVDF membranes. After blocking with BSA, membranes were incubated with the corresponding primary antibodies at 4 °C overnight. Following washing with TBST, membranes were incubated with horseradish peroxidase-conjugated anti-rabbit IgG or anti-mouse IgG (1:2000; Cell Signaling Technology, USA). Protein bands were detected and analyzed using a Tanon 5200 system (China) and ECL reagent (Millipore, USA).

Primary antibody dilutions were as follows: BDNF (1:4000), Bcl-2 (1:30000), CGRP (1:1000), SP (1:500), p-CaMKII (1:1000), CaMKII (1:2000), p-CREB (1:1000), CREB (1:1000), p-JAK1 (1:1000), JAK1 (1:1000), p-STAT1 (1:1000), STAT1 (1:1000), and GAPDH (1:100000).

## 1.18. Establishment of the rat periodontitis alveolar bone defect model and implant placement

All animal experiments were performed in accordance with the ethical guidelines established by Guangdong Huawei Testing Co., Ltd. (Approval No. 202509003).

For periodontitis induction, rats were anesthetized and 3-0 silk ligatures were placed subgingivally around the bilateral maxillary first molars (M1) without damaging the adjacent gingival tissue. After 8 weeks of ligation, the ligatures were removed and the periodontitis model was confirmed. For implant surgery, rats were anesthetized and the bilateral maxillary first molars were extracted. The extraction sockets were irrigated with sterile saline. A 1 mm diameter drill was used to prepare the implant site in the palatal root socket region under continuous saline irrigation. Finally, the hydrogel was injected into the extraction socket followed by placement of a 1.0 mm × 3 mm titanium alloy implant screw.

**Ultrasound intervention protocol:** Beginning 24 h post-implantation, the ultrasound stimulation group received daily transcutaneous ultrasound irradiation over the implant region (1.0 MHz, 1.5 W cm⁻², 50% duty cycle, 10 min per session) for 14 consecutive days. From day 15 onward, ultrasound was discontinued, and animals were observed until week 8. This protocol was designed so that the 2-week ultrasound intervention window aligned with both the effective piezo-functional maintenance period of the hydrogel and the temporal macrophage M1→M2 polarization switch.

Groups: CTRL, Periodontitis, Gel, CN/rGO, CN/rGO + US, CN/rGO + US + TP1L.

## 1.19. In vivo biocompatibility and antibacterial performance

**Hemocompatibility testing:** Hemolysis rates were evaluated by co-incubating the hydrogels with rat red blood cells (RBCs). PBS and Triton X-100 served as negative and positive controls, respectively. Hemolysis percentages were calculated based on supernatant absorbance at 540 nm.

**Subcutaneous implantation:** To assess biodegradability and overall biocompatibility, hydrogels were implanted subcutaneously in healthy rats. Implants were retrieved at predetermined time points, photographed, and weighed to evaluate residual mass.

**Histopathological examination of major organs:** At the osseointegration study endpoint (8 weeks), major organs (heart, liver, spleen, lungs, and kidneys) were harvested, fixed, sectioned, and stained with hematoxylin and eosin (H&E) to screen for potential systemic toxicity.

**Antibacterial performance:** At 4 weeks post-implantation, implants were carefully retrieved under aseptic conditions (n = 3 per group). Implants were sonicated in PBS to detach adherent bacteria, and the resulting suspensions were serially diluted and plated on agar for CFU counting after culture.

## 1.20. Micro-CT analysis

At weeks 4 and 8, corresponding experimental rats were euthanized, the maxillae were harvested, and specimens were fixed in 4% paraformaldehyde for 48 h. Scanning was performed using a micro-computed tomography (micro-CT) system (Tomo NT, Skyscan, Antwerp, Belgium). Files were reconstructed using a modified Feldkamp algorithm and analyzed with micro-tomography analysis software (Tomo NT). Quantitative parameters including peri-implant bone volume fraction (BV/TV) and bone mineral density (BMD) were calculated.

## 1.21. Histological analysis

Peri-implant bone tissue was harvested at 1 week post-surgery, total RNA was extracted, and mRNA expression levels of local inflammatory cytokines (IL-6, TNF-α, IL-1β) were analyzed by qPCR.

**Immunofluorescence staining:** Frozen sections of peri-implant tissues collected at weeks 1 and 2 were stained with anti-CD86 (M1), anti-CD206 (M2), anti-iNOS, and anti-Arg-1 antibodies to assess macrophage polarization. Sections collected at week 4 were stained with anti-CGRP (sensory nerve marker) and anti-CD31 (endothelial cell marker) antibodies to evaluate neurovascular regeneration. Fluorescence images were acquired and quantified.

Maxillary specimens were immersed in 4% paraformaldehyde, decalcified in 10% EDTA for 1 week, embedded in paraffin, and sectioned. Bone defect samples were stained with H&E and Masson’s trichrome and observed under a light microscope (CX21, Olympus, Tokyo, Japan).

## 1.22. Statistical analysis

All quantitative data are expressed as mean ± standard deviation (SD). Comparisons between two groups were performed using Student’s *t*-test, while multi-group comparisons were conducted using one-way analysis of variance (ANOVA) followed by Tukey’s post hoc test. In vitro experiments were independently repeated at least three times (specific sample sizes are indicated in each figure legend); in vivo experiments: *n* = 4 per group. Data analysis was performed using GraphPad Prism 9.0 software. **p* < 0.05, ***p* < 0.01, ****p* < 0.001, *****p* < 0.0001; ns = not significant.

## Reference

[1] H. Teixeira, A.C. Branco, I. Rodrigues, D. Silva, S. Cardoso, R. Colaço, A.P. Serro, C.G. Figueiredo-Pina, Effect of albumin, urea, lysozyme and mucin on the triboactivity of Ti6Al4V/zirconia pair used in dental implants, Journal of the Mechanical Behavior of Biomedical Materials 118 (2021), 10.1016/j.jmbbm.2021.104451.

[2] M.L. Sun, J.M. Wang, X.B. Huang, R.Q. Hang, P.D. Han, J.Q. Guo, X.H. Yao, P.K. Chu, X.Y. Zhang, Ultrasound-driven radical chain reaction and immunoregulation of piezoelectric-based hybrid coating for treating implant infection, Biomaterials 307 (2024), 10.1016/j.biomaterials.2024.122532.

[3] S. Vaidya, M. Joshi, S. Ghosh, N. More, R. Velyutham, S.S. Babu, G. Kapusetti, Bioactive ZnO Decorated PVDF-Based Piezoelectric, Osteoconductive Nanofibrous Coatings for Orthopedic Implants, Journal of Biomedical Materials Research Part A 113(8) (2025), 10.1002/jbm.a.37971.

[4] C.F. Wang, W.C. Sun, Y.M. Xiang, S.L. Wu, Y.F. Zheng, Y. Zhang, J. Shen, L. Yang, C.Y. Liang, X.M. Liu, Ultrasound-Activated Piezoelectric MoS2 Enhances Sonodynamic for Bacterial Killing, Small Science 3(7) (2023), 10.1002/smsc.202300022.

[5] J.T. Xie, Z.H. Zhang, Z.H. Yu, B.X. Sun, Y.X. Yang, G.Y. Wang, C.J. Shuai, Ultrasound-Activated BiOI/Ti3C2 Heterojunctions in 3D-Printed Piezocatalytic Antibacterial Scaffolds for Infected Bone Defects, Materials 18(15) (2025), 10.3390/ma18153533.

[6] Z. Yang, M. Yuan, B. Liu, Z.Z. Ma, J. Ma, X.Y. Ma, K. Li, P.A. Ma, Z.Y. Cheng, J. Lin, Dual-Defect Regulated G-C3N4 for Piezoelectric Catalytic Tumor Therapy with Enhanced Efficacy, Advanced Materials 37(11) (2025), 10.1002/adma.202412069.

**Table S1. PCR primer sequences.**

| Gene | Primer sequence |
| --- | --- |
| IL-6-F | 5'-TAGTCCTTCCTACCCCAATTTCC-3' |
| IL-6-R | 3'-TTGGTCCTTAGCCACTCCTTC-5' |
| TNF-α-F | 5'-GACGTGGAACTGGCAGAAGAG-3' |
| TNF-α-R | 3'-TTGGTGGTTTGTGAGTGTGAG-5' |
| IL-1β-F | 5'-ACAGGCTCCGAGATGAACAA-3' |
| IL-1β-R | 3'-GGCCACAGGTATTTTGTCGT-5' |
| IL-10-F | 5'-GCATGGCCCAGAAATCAAGG-3' |
| IL-10-R | 3'-GAGAAATCGATGACAGCGCC-5' |
| Arg-1-F | 5'-ATGGGCAACCTGTGTCCTTT-3' |
| Arg-1-R | 3'-CTACGTCTCGCAAGCCAATG-5' |
| ALP-F | 5'-TCCGTGGGCATTGTGACTAC-3' |
| ALP-R | 3'-TGGTGGCATCTCGTTATCCG-5' |
| OCN-F | 5'-AGCAAGAAACTCTTCCAAGCAA-3' |
| OCN-R | 3'-GTGAGATTCGTCAGATTCATCCG-5' |
| RUNX2-F | 5'-GACTGTGGTTACCGTCATGGC-3' |
| RUNX2-R | 3'-ACTTGGTTTTTCATAACAGCGGA-5' |
| BMP-2-F | 5'-TTGGACACCAGGTTAGTGAATCA-3' |
| BMP-2-R | 3'-TCTCCTCTAAATGGGCCACTT-5' |
| PDGF-BB-F | 5'-CATCCGCTCCTTTGATGATCTT-3' |
| PDGF-BB-R | 3'-GTGCTCGGGTCATGTTCAAGT-5' |
| ET-1-F | 5'-GAGCGCGTCGTACCGTATG-3' |
| ET-1-R | 3'-ACTGACATCTAACTGCCTGGT-5' |
| GAPDH-F | 5'-TTCCAGGAGCGAGACCCCACTA-3' |
| GAPDH-R | 3'-GGGCGGAGATGATGACCCTTTT-5' |

**Table S2. Comparison of piezoelectric materials.**

| Piezoelectric system (active phase) |  | Form/formulation | Activation & key piezo-mechanism | Anti-bacterial | Immunomodulation | Osteo- /angiogenesis | Neurogenesis | Metal-free | Ref. |
| --- | --- | --- | --- | --- | --- | --- | --- | --- | --- |
| BaTiO₃  (BaTiO₃₋ₓ / L-arginine) |  | Nanorod-array hybrid coating on Ti implant | US-triggered; intrinsic ferroelectric perovskite piezoelectricity drives sonodynamic ROS + RNS and M2 immunoregulation | ✓ | ✓ | ✓ | — | — | [2] |
| ZnO  (ZnO / PVDF) |  | Electrospun piezoelectric nanofibrous coating on Ti implant | Mechanical/US strain; wurtzite ZnO reinforces PVDF β-phase piezoelectricity; Zn²⁺-mediated antibacterial, osteoconductive | ✓ | — | ✓ | — | — | [3] |
| MoS₂  (MoS₂ / Cu₂O) |  | Heterojunction nanocomposite (dispersion) | US-triggered; 2D MoS₂ piezo-polarization + heterojunction charge transfer; sonodynamic ROS and GSH depletion | ✓ | — | — | — | — | [4] |
| Bismuth oxyhalide  (BiOI·BiOCl family; BiOI / Ti₃C₂) |  | 3D-printed PCL piezocatalytic scaffold | US-triggered; layered bismuth-oxyhalide piezocatalyst (d₃₃ ≈ 25 pm/V) + Schottky heterojunction → ROS | ✓ | — | ✓ | — | — | [5] |
| g-C₃N₄  (defect-engineered) |  | Metal-free 2D nanosheets (dispersion) | US-triggered; defect-regulated g-C₃N₄ piezocatalytic ROS (demonstrated in a tumor model) | △ | — | — | — | ✓ | [6] |
| This work  CN/rGO (g-C₃N₄ / rGO) |  | Injectable conductive piezoelectric hydrogel (PVA/tsPBA + PEDOT) | US-triggered; metal-free g-C₃N₄/rGO Schottky-contact heterostructure; persistent piezoelectric field + piezocatalytic ROS (apparent d₃₃ ≈ 20 pm/V) | ✓ | ✓ | ✓ | ✓ | ✓ | This work |

# Supplementary Figures

Figure S1. Optimization of CN/rGO heterojunction composition. Performance comparison of CN/rGO heterojunctions with different rGO contents (10, 15, and 20 wt%). (A) Efficiency of ·OH-mediated MB degradation catalyzed by each heterojunction formulation under ultrasound irradiation (1.0 MHz, 1.5 W cm⁻², 50% duty cycle, 5 min) (ROS generation capacity). (B) EIS Nyquist plots of each heterojunction formulation measured in 0.1 M Na₂SO₄ electrolyte. The 15 wt% rGO group exhibited optimal ROS catalytic efficiency and the lowest charge-transfer resistance. n = 3.

Figure S2. Effect of CN/rGO heterojunction concentration on ROS generation capacity. Residual percentage of MB absorbance intensity (664 nm) after co-incubation with CN/rGO heterojunction dispersions at different concentrations under ultrasound irradiation (1.0 MHz, 1.5 W cm⁻², 50% duty cycle, 5 min).

Figure S3. Optimization of CN/rGO loading concentration in the hydrogel. Comprehensive screening of hydrogels with different CN/rGO contents (0.25, 0.5, and 1.0 wt%). (A) Efficiency of ·OH-mediated MB degradation under ultrasound irradiation (1.0 MHz, 1.5 W cm⁻², 50% duty cycle, 5 min) (ROS generation capacity). (B) CCK-8 assay of RAW 264.7 macrophage viability after 3 days of co-culture with hydrogels of different CN/rGO contents. The 0.5 wt% group represents the optimal balance between functional efficacy and biosafety. n = 3. *p < 0.05, **p < 0.01.

Figure S4. Strain-amplitude sweep of the CN/rGO hydrogel. Strain-amplitude sweep test of the CN/rGO hydrogel at 37 °C. Strain range: 0.01%–100%; frequency: 10 Hz. G′ consistently exceeded G′′, confirming stable gel-state behavior.

Figure S5. Steady-state shear test of the CN/rGO hydrogel. Viscosity of the CN/rGO hydrogel as a function of shear rate (0.1–10 s⁻¹) at 37 °C. Viscosity decreased markedly with increasing shear rate, confirming shear-thinning behavior.

Figure S6. Temporal changes in piezo-catalytic activity during CN/rGO hydrogel degradation. (A) CN/rGO hydrogels were immersed in lysozyme-containing PBS (1 mg/mL, 37 °C) to simulate in vivo degradation. Samples were collected at days 1, 3, 7, and 14 to assess MB degradation efficiency under ultrasound irradiation (1.0 MHz, 1.5 W cm⁻², 50% duty cycle, 5 min). Data were normalized to day 1 (set as 100%). The right Y-axis shows the residual hydrogel mass curve, illustrating the correspondence between degradation progression and catalytic activity attenuation. n = 3. (B) Comparison of piezoelectric current output before and after hydrogel degradation. Piezoelectric short-circuit current of CN/rGO hydrogels (immersed in lysozyme-containing PBS, 37 °C) at days 1 and 14 under ultrasound (1.0 MHz, 1.5 W cm⁻², 50% duty cycle). Serves as direct piezoelectric verification of the catalytic activity data in Fig. S7A. n = 3. *p < 0.05, **p < 0.01.

Figure S7. Verification of CN/rGO hydrogel electrical conductivity. A light-emitting diode (LED) illuminated within a circuit incorporating the CN/rGO piezoelectric conductive hydrogel, visually confirming its electrical conductivity.

Figure S8. Hemolysis assay. Hemolysis evaluation of gel and CN/rGO hydrogels. (A) Photographs of the hemolysis assay. (B) Quantitative analysis of hemolysis rates. PBS and Triton X-100 served as negative and positive controls, respectively. No significant difference in hemolysis rate was observed between the hydrogel groups and the PBS group.

Figure S9. Subcutaneous implantation experiment. In vivo degradation of hydrogels (gel, g-CN, g-CN/rGO, g-CN/rGO + US) after subcutaneous implantation. (A–C) Gross photographs of implants at different time points. (D) H&E staining of residual hydrogel.

Figure S10. H&E staining of major organs. H&E staining of major organs (heart, liver, spleen, lung, and kidney) of rats at 8 weeks after CN/rGO hydrogel implantation. No apparent histological damage was observed in any group.

Figure S11. Macrophage biocompatibility. CCK-8 assay of RAW 264.7 macrophage viability after 3 days of co-culture with different hydrogels. n ≥ 3. *p < 0.05.

Figure S12. Quantification of macrophage polarization immunofluorescence. Fluorescence intensity quantification of iNOS (M1) and Arg-1 (M2) immunofluorescence co-staining in RAW 264.7 macrophages: (A) Day 3, (B) Day 7. n ≥ 3. *p < 0.05, **p < 0.01.

Figure S13. Cytoskeletal morphology of PC12 neurons. FITC-phalloidin/DAPI cytoskeletal staining of PC12 neurons in different treatment groups under LPS stimulation, showing adhesion morphology and spreading area.

Figure S14. PC12 neuron viability. CCK-8 cell viability assay of PC12 neurons in different treatment groups after 3 days of culture under LPS stimulation. n ≥ 3. *p < 0.05, **p < 0.01.

Figure S15. Live/dead staining of PC12 cells. Calcein-AM/PI live/dead staining of PC12 cells after 1 day of co-culture under LPS stimulation. Green: live cells (Calcein-AM); red: dead cells (PI).

Figure S16. Western blot analysis of BDNF and Bcl-2 protein expression levels in PC12 cells.

Figure S17. Transwell migration assay of PC12 cells. Transwell assay evaluating the effect of different hydrogel extracts on PC12 cell migration. (A) Representative images of crystal violet-stained migrated cells. (B) Quantification of migrated cell numbers. n = 3. *p < 0.05, **p < 0.01.

Figure S18. Quantitative analysis of Western blot results for p-CaMKII, p-CREB, CGRP, and SP protein expression in PC12 cells. Cells were cultured with conditioned medium from different groups, with or without the CaMKII inhibitor KN-93 or the CREB inhibitor KG-501. n = 3. *p < 0.05, **p < 0.01.

Figure S19. Live/dead staining of HUVECs. Calcein-AM/PI live/dead staining of HUVECs after 3 days of culture in different treatment groups under LPS stimulation. Green: live cells; red: dead cells.

Figure S20.HUVEC viability. CCK-8 cell viability assay of HUVECs after 24 h of culture in different treatment groups under LPS stimulation. n ≥ 3. *p < 0.05, **p < 0.01.

Figure S21. HUVEC cytoskeletal morphology. FITC-phalloidin/DAPI cytoskeletal staining of HUVECs in different treatment groups under LPS stimulation, showing adhesion morphology.
